# Activation of Neurotoxic Astrocytes Due to Mitochondrial Dysfunction Triggered by *POLG* Mutation

**DOI:** 10.7150/ijbs.93445

**Published:** 2024-05-11

**Authors:** Kristina Xiao Liang, Anbin Chen, Atefeh Kianian, Cecilie Katrin Kristiansen, Tsering Yangzom, Jessica Furriol, Lena Elise Høyland, Mathias Ziegler, Torbjørn Kråkenes, Charalampos Tzoulis, Evandro Fei Fang, Gareth John Sullivan, Laurence A. Bindoff

**Affiliations:** 1 Department of Clinical Medicine (K1), University of Bergen, Jonas Lies vei 87, P. O. Box 7804, 5021 Bergen, Norway.; 2 Neuro-SysMed, Center of Excellence for Clinical Research in Neurological Diseases, Haukeland University Hospital, Jonas Lies vei 87, P. O. Box 7804, 5021 Bergen, Norway.; 3 Department of Neurosurgery, Xinhua Hospital Affiliated toShanghai Jiaotong University School of Medicine, No. 1665, Kongjiang Road, 200092 Shanghai, China.; 4 Department of Medicine, Haukeland University Hospital, Jonas Lies vei 87, P. O. Box 7804, 5021 Bergen, Norway.; 5 Department of Biomedicine, University of Bergen, Jonas Lies Vei 91, 5009 Bergen, Norway.; 6 Department of Clinical Molecular Biology, University of Oslo and Akershus University Hospital, 1478 Oslo, Norway.; 7 The Norwegian Centre on Healthy Ageing (NO-Age), 1478 Oslo, Norway.; 8 Department of Molecular Medicine, Institute of Basic Medical Sciences, University of Oslo, P. O. Box 1105, 0317 Oslo, Norway.; 9 Institute of Immunology, Oslo University Hospital, P.O. Box 4950, 0424 Oslo, Norway.; 10 Hybrid Technology Hub Centre of Excellence, Institute of Basic Medical Sciences, University of Oslo, P. O. Box 1110, 0317 Oslo, Norway.; 11 Department of Pediatric Research, Oslo University Hospital, P. O. Box 4950, 0424 Oslo, Norway.

## Abstract

Mitochondrial diseases are associated with neuronal death and mtDNA depletion. Astrocytes respond to injury or stimuli and damage to the central nervous system. Neurodegeneration can cause astrocytes to activate and acquire toxic functions that induce neuronal death. However, astrocyte activation and its impact on neuronal homeostasis in mitochondrial disease remain to be explored. Using patient cells carrying *POLG* mutations, we generated iPSCs and then differentiated these into astrocytes. POLG astrocytes exhibited mitochondrial dysfunction including loss of mitochondrial membrane potential, energy failure, loss of complex I and IV, disturbed NAD^+^/NADH metabolism, and mtDNA depletion. Further, POLG derived astrocytes presented an A1-like reactive phenotype with increased proliferation, invasion, upregulation of pathways involved in response to stimulus, immune system process, cell proliferation and cell killing. Under direct and indirect co-culture with neurons, POLG astrocytes manifested a toxic effect leading to the death of neurons. We demonstrate that mitochondrial dysfunction caused by *POLG* mutations leads not only to intrinsic defects in energy metabolism affecting both neurons and astrocytes, but also to neurotoxic damage driven by astrocytes. These findings reveal a novel role for dysfunctional astrocytes that contribute to the pathogenesis of POLG diseases.

## Background

In an increasingly aging global population, neurodegeneration is now a leading threat to human health. Understanding the process of neurodegeneration is changing from a purely neuron-centric view to a more comprehensive perspective in which several cell types interact to drive this process, and where astrocytes, previously considered only supportive, have moved center stage 1-5. Despite advances, mechanistic understanding remains constrained within paradigms such as oxidative stress, proteasome impairment, accumulation of abnormal protein aggregates and mitochondrial dysfunction 6, 7. Of these, mitochondrial dysfunction is a recurring theme, particularly in common diseases 8-10; such as Parkinson's (PD) and Alzheimer's (AD) diseases that together affect ~10% of population, and diabetic retinopathy, the most common form of blindness in working aged people 11, 12. How a mitochondrial defect leads to tissue destruction and subsequent loss of functional neurons remains, however, unclear.

Of interest in the context of mitochondrial dysfunction are proteins involved in mitochondrial DNA replication (mtDNA), particularly polymerase gamma (pol γ) as the enzyme that replicates and repairs mtDNA 13. Mutations in the *POLG* gene, which encodes the catalytic subunit of polymerase γ, are the primary cause of inherited mitochondrial diseases. These mutations, along with variations in mtDNA, significantly affect mitochondrial function. Clinically, these mutations cause a continuum of overlapping phenotypes from infantile to adult disorders 14. At the molecular level, mutations in *POLG* lead to mtDNA maintenance defects and mitochondrial dysfunction. Interestingly, the molecular mtDNA defect differs depending on the tissue; multiple deletions occur in skeletal muscle while neurons and hepatocytes show mtDNA depletion 15. The underlying mechanisms behind decreased mtDNA quality and mitochondrial dysfunction in POLG related disease remain, however, elusive. In post-mortem studies, we have shown that the loss of neurons in POLG related disease is driven by severe mtDNA depletion 15.

The contribution of glial cell dysfunction to neurodegenerative disease has come more into focus 3, 16-18. Astrocytes are both highly abundant 19 and play a crucial role in the support and modulation of neuronal function including regulating glutamate turnover, ion and water homeostasis, synapse formation/modulation, tissue repair, energy storage, and defense against oxidative stress 19, 20. These cells are also critical for neuronal metabolism 21, 22 and are a major component of neurovascular coupling 23. Greater understanding of the importance of astrocytes has shifted focus to the role of these cells in neurological diseases such as PD, AD 24, 25, Huntington's disease (HD) 26 and amyotrophic lateral sclerosis (ALS) 27. However, the role of astrocytes in mitochondrial diseases such as POLG has yet to be explored.

Both disease and cerebral injury can induce astrocytes to enter a 'reactive' state in which gene expression changes markedly 3. These reactive astrocytes are mainly divided into A1 and A2 types according to their function: LPS-induced neuroinflammation A1 astrocytes lose their supportive role and acquire toxic functions mediated by secreted neurotoxins, thereby inducing neurons and oligodendrocytes die rapidly; Ischemia-induced A2-reactive astrocytes promote neuronal survival and tissue repair. Recent studies suggested that activated microglia induced astrocyte conversion to the A1 reactive phenotype by releasing interleukin 1 alpha (IL1α), tumor necrosis factor alpha (TNFα), and the complement component subunit 1q (C1q) 3. In neurodegenerative states such as AD 28, PD 29, multiple sclerosis (MS) 28 and ALS 17. Reactive astrocytes can display both neuroprotective and neurodegenerative functions. The role of astrocyte reactivation and the consequences this has for neuronal homeostasis in mitochondrial disease and mitochondrial dysfunction has not been explored.

Stem cell technologies, including induced pluripotent stem cells (iPSCs) and multicellular organoid models are revolutionizing our ability to investigate complex systems and human disease 17, 30, 31. Our aim in this study was to use a human stem cell-based culture system to examine the astrocytic contribution to POLG related disease of the brain and investigate whether mitochondrial dysfunction would stimulate astrocytes to become toxic for neurons. To achieve this, we generated iPSC-derived astrocytes from two patients harboring *POLG* mutations. Using cellular, metabolic, and transcriptomic approaches, we found that *POLG* mutations not only cause intrinsic defects in energy metabolism affecting neurons and astrocytes, but also astrocyte-driven neurotoxic damage. Here, we describe the mitochondrial profile of iPSC-derived astrocytes from POLG related diseases, validating this model as a powerful tool for studying disease mechanisms and for non-invasive drug-targeting assays in vitro. Our findings reveal novel roles for dysfunctional astrocytes that contribute to the pathogenesis of mitochondrial diseases, which provide a novel disease target.

## Materials and methods

### Derivation of iPSCs and neural stem cells (NSCs) generation

Human iPSCs were reprogrammed from fibroblasts as described in our previous publication 31, 32. As shown in Supplementary Table 1, two POLG patients derived iPSCs were used in this study, including one homozygous c.2243G>C, p.W748S/W748S (WS5A) and one compound heterozygous c.1399G>A/c.2243G>C, p.A467T/W748S (CP2A). A panel of control were used in this study, Detroit 551 (ATCC^®^ CCL 110^™^), AG05836B fibroblasts (RRID: CVCL_2B58) and two human embryonic stem cell (ESC) lines: HS429 and HS360.

All iPSC and ESC lines were maintained in E8 medium (Invitrogen, A1517001) on Geltrex (Invitrogen, A1413302) coated 6-well plate (Thermo Scientific, 140675).

NSCs were generated from the POLG and control iPSC lines by lifting cells into a Chemically Defined Medium (CDM) with epidermal growth factor (EGF) and fibroblast growth factor-2 (FGF-2) both at 100 ng/ml, as described previously 32.

### Astrocyte differentiation

iPSC-derived NSCs were placed on poly-D-lysine (PDL) coated coverslips (Neuvitro, GG-12-15-PDL). The following day, the cells were changed into astrocyte differentiation medium, as described in Supplementary Table 1. The medium was changed every other day for the first week, every two days for the second week and every three days for the third and fourth week. After 28 days of differentiation, the cells were cultured in maturation medium AGM^TM^ Astrocyte Growth Medium BulletKit^TM^ (Lonza, CC-3186) as described in Supplementary Table 2, for one more month.

### Dopaminergic neuron (DA neuron) differentiation

iPSC-derived neurospheres which were generated from 5 days' neural induction, were maintained in CDM supplemented with 100 ng/ml FGF8b (R&D systems, 423-F8) over a period of 7 days to initiate DA neuron progenitor induction. The following 7 days, the medium was changed to CDM supplemented with 1 µM purmorphamine (PM) (EMD Millipore, 540220-5MG) and 100 ng/ml FGF8b. Termination of the suspension cultures was performed by dissociating the spheres into single cells by incubation with TrypLE^™^ Express followed by trituration and subsequent plating into monolayers. The DA neurons were matured in DA medium: CDM supplemented with 10 ng/ml BDNF (PeproTech, 450-02) and 10 ng/ml GDNF (PeproTech, 450-10) on Poly-L-Ornithine (Sigma-Aldrich, P4957) and laminin (Sigma-Aldrich, L2020) coated plates.

### Immunocytochemistry and immunofluorescence (ICC/IF) staining

Cells were fixed with 4% (v/v) paraformaldehyde (PFA, VWR, 100503-917) and blocked using blocking buffer containing 10% (v/v) normal goat serum (Sigma-Aldrich, G9023) with 0.3% (v/v) Triton^™^ X-100 (Sigma-Aldrich, X100-100ML). The cells were then incubated with primary antibody solution overnight at 4°C and further stained with secondary antibody solution (1:800 in blocking buffer) for 1 hour (h) at room temperature (RT). NSCs were stained with rabbit anti-PAX6, anti-NESTIN, anti-SOX2.Mitochondrial respiratory chain complex I subunit were stained with anti-NDUFB10. Astrocytes and oligodendrocytes were stained with anti-GFAP, anti-S100β, anti-EAAT-1, anti-GS and anti- DCX, respectively. The antibodies used for DA neuron staining were anti-TH, anti-TuJ1 and anti-MAP2. The secondary antibodies used were Alexa Fluor^®^ goat anti-rabbit 488 Alexa Fluor^®^ goat anti-mouse 594 and Alexa Fluor^®^ goat anti-chicken 594. After incubation with secondary antibodies, the coverslips were mounted onto cover slides using prolong diamond antifade mounting medium with DAPI (Invitrogen, P36962). Details of the antibodies used were listed in Key Resources Table.

For staining of neurospheres, spheres were spread directly onto cover slides and left at RT until completely dry and then fixed with 4% (v/v) PFA. After two washes with PBS, the spheres were covered in PBS with 20% sucrose, sealed with parafilm, and incubated overnight at 4°C. The spheres were blocked with blocking buffer for 2 hours at RT and the primary antibodies were added to the samples overnight at 4°C. After washing the samples for 3 hours in PBS with a few changes of buffer, incubation with secondary antibodies (as described above) was conducted overnight at 4°C in a humid and dark chamber. Coverslips were mounted using Fluoromount G^®^ (Southern Biotech, 0100-01) before imaging was performed using the Leica TCS SP5 or SP8 STED confocal microscope (Leica Microsystems, Germany).

### Mitochondrial volume and mitochondrial membrane potential (MMP) measurement

To measure mitochondrial volume and MMP, cells were double stained with 150 nM MitoTracker Green (MTG) and 100 nM Tetramethylrhodamine Ethyl Ester (TMRE) for 45 min at 37°C. Cells treated with 100 µM Carbonyl cyanide-p-trifluoromethoxyphenylhydrazone (FCCP) (Abcam, ab120081) were used as negative control. Stained cells were washed with PBS, detached with TrypLE^™^ Express and neutralized with media containing 10% FBS. The cells were immediately analyzed on a FACS BD Accuri^™^ C6 flow cytometer (BD Biosciences, San Jose, CA, USA). The data analysis was performed using Accuri™ C6 software.

### L-lactate production measurement

L-lactate generation was analyzed by colorimetric L-lactate assay kit (Abcam, ab65331) according to the manufacturer's instructions. Endpoint lactate concentration was determined in a 96-well plate by measuring the initial velocity (2 min) of the balance between NAD^+^ and NADH by lactate dehydrogenase. Immediately following the extracellular flux assay, the plate was measured at OD 450 nm in a microplate reader (VICTOR^™^ XLight, PerkinElmer).

### Intercellular and mitochondrial reactive oxygen species (ROS) production

Intracellular ROS production was measured by flow cytometry using dual staining of 30 µM 2′,7′-Dichlorodihydrofluorescein diacetate (DCFDA) (Abcam, ab11385) and 150 nM MitoTracker Deep Red (MTDR) (Invitrogen, M22426), which enabled us to assess ROS level related to mitochondrial volume. Mitochondrial ROS production was quantified using co-staining of 10 µM MitoSOX^™^ Red Mitochondrial Superoxide Indicator (Invitrogen, M36008) and 150 nM MTG to evaluate ROS level in relation to mitochondrial volume. Stained cells were detached with TrypLE^™^ Express and neutralized with media containing 10% FBS. The cells were immediately analyzed on a FACS BD Accuri^™^ C6 flow cytometer.

### NADH metabolism and ATP measurement using Liquid Chromatography Mass Spectrometry (LC-MS) analysis

Cells were washed with PBS and extracted by addition of ice-cold 80% methanol followed by incubation at 4°C for 20 min. Thereafter, the samples were stored at -80°C overnight. The following day, samples were thawed on a rotating wheel at 4°C and subsequently centrifuged at 16000 g at 4°C for 20 min. The supernatant was added to 1 volume of acetonitrile and the samples were stored at -80°C until analysis. The pellet was dried and subsequently reconstituted in a lysis buffer (20 mM Tris-HCl (pH 7.4), 150 mM NaCl, 2% SDS, 1 mM EDTA) to allow for protein determination with BCA protein assay (Thermo Fisher Scientific, 23227).

Separation of the metabolites was achieved with a ZIC-pHILC column (150 x 4.6 mm, 5 μm; Merck) in combination with the Dionex UltiMate 3000 (Thermo Scientific) liquid chromatography system. The column was kept at 30°C. The mobile phase consisted of 10 mM ammonium acetate pH 6.8 (Buffer A) and acetonitrile (Buffer B). The flow rate was kept at 400 µL/min and the gradient was set as follows: 0 min 20% Buffer B, 15 min to 20 min 60% Buffer B, 35 min 20% Buffer B. Ionization was subsequently achieved by heated electrospray ionization facilitated by the HESI-II probe (Thermo Scientific) using the positive ion polarity mode, and a spray voltage of 3.5 kV. The sheath gas flow rate was 48 units with an auxiliary gas flow rate of 11 units, and a sweep gas flow rate of 2 units. The capillary temperature was 256°C and the auxiliary gas heater temperature was 413°C. The stacked-ring ion guide (S-lens) radio frequency (RF) level was at 90 units. Mass spectra were recorded with the Q Exactive mass spectrometer (Thermo Scientific) and data analysis was performed with the Thermo Xcalibur Qual Browser. Standard curves generated for NAD^+^ and NADH were used as references for metabolite quantification.

### Flow cytometric measurement of mitochondrial complexes levels

Cells were detached with TrypLE^™^ Express, pelleted and fixed in 1.6% (v/v) PFA at RT for 10 minutes, before permeabilization with ice-cold 90% methanol. The cells were blocked using a buffer containing 0.3M glycine, 5% goat serum and 1% bovine serum albumin (BSA) in PBS. For staining of mitochondrial respiratory chain complexes, primary antibody anti-NDUFB10, anti-COX IV and anti-SDHA were added, followed by secondary antibody incubation. The cells were immediately analyzed on a BD Accuri^™^ C6 flow cytometer. Details of the antibodies used were listed in Key Resources Table.

### Complex I enzyme activity measurement

Complex I enzyme activity was measured using Complex I Enzyme Activity Microplate Assay Kit (Abcam, ab109721) according to the manufacturer's protocol. Briefly, cells were seeded in six-well plates at a density of 200000 cells per well for 48 hours. The cell lysate was harvested, loaded into a 96-well microplate, and incubated for 3 hours. After washing three times with buffer, assay solution was added, and the reaction was measured using a microplate reader (Infinite F50) with a wavelength of 450 nm for 60 min. Activity is expressed as the change in absorbance per minute (OD/min) per 800 μg/ml.

### Indirect co-culture system

The transwell chambers with pore size 0.4 µm (Corning, 3450) were used for astrocyte-neural indirect co-culture experiments. iPSC-derived astrocytes were plated onto the insert membranes, while normal iPSC-derived DA neurons were seeded on the bottom of each plate respectively.

### Cell proliferation and viability assay

A total number of 10000 cells were seeded in 96-well plates and cultured for 6 days. Cell numbers were counted, and cell viability was determined near trypan blue staining, where unstained cells were defined as viable cells.

### Apoptosis detection by flow cytometry

Apoptotic cell death was measured by flow cytometry using the Annexin V-FITC/PI double staining kit (Invitrogen, V13242) according to manufacturer's instructions. The number of viable (Annexin negative/PI negative), early apoptotic (Annexin positive/PI negative), and late apoptotic/necrotic (Annexin and PI positive) cells were determined using Accuri^™^ C6 software.

### Wound healing assay

A total number of 5000 cells was seeded into an Ibidi culture-insert chamber (Ibidi, 81176). After the cells reached 100% confluence, the insert was removed and cultured for a further 24hours. Cells were observed and images were captured after 0 h, 4 hours and 24 hours.

### Western blot analysis

Extraction of protein was done using 1X RIPA lysis and extraction buffer (Sigma-Aldrich, R0278) supplemented with Halt^™^ Protease and Phosphatase Inhibitor Cocktail (Invitrogen, 78444). Protein concentration was determined using BCA protein assay. The cell protein was loaded into NuPAGE^™^ 4-12% Bis-Tris Protein Gels (Invitrogen, NP0321PK2), and resolved in PVDF membrane (Bio-Rad, 1704157) using the Trans-Blot^®^ Turbo^™^ Transfer System (Bio-Rad, Denmark). Membranes were blocked with 5% non-fat dry milk or 5% BSA in TBST for 1 hour at RT. Membranes were then incubated overnight at 4°C with anti-UCP2, anti-GFAP, anti-GS, anti-Phospho-SIRT1 (Ser47), anti-SIRT3 (D22A3), anti-β-catenin, anti-N-cadherin, anti-C3 and anti-GAPDH antibody conjugated to horseradish peroxidase (HRP) as a control. After washing in TBST, membranes were incubated with donkey anti-mouse antibody or swine anti-rabbit antibody conjugated to HRP secondary for 1 hour at RT. Super signal west Pico chemiluminescent substrate (Thermo Fisher Scientific, 34577) was used as enzyme substrate according to manufacturer's recommendations. The membranes were visualized by ChemiDoc imaging systems (BioRad). Details of the antibodies used were listed in Key Resources Table.

### Tissue studies

Post-mortem examination was performed on brain tissue from 4 POLG patients, including the prefrontal cortex, occipital cortex, cerebellum, and spinal cord. The *POLG* mutations for these patients are 2 patients (AT-1A/44yrs, female and AT-1B/47yrs, male) with A467T/A467T mutation; 2 patients (WS-1A/41yrs, female and WS-3A/43yrs, female) with W748S/W748S mutation. Informed consent was obtained from all subjects and that the experiments conformed to the principles set out in the WMA Declaration of Helsinki and the Department of Health and Human Services Belmont Report. Hematoxylin-eosin (HE) staining was performed by standard procedures. Immunohistochemistry was performed using antibodies against GFAP, an astrocytic marker, and HLA-DR/DP/DQ, a microglial marker. Antibodies were obtained from DAKO, Glostrup, Denmark.

### RNA sequencing

Total RNA was extracted using QIAGEN RNeasy Kit (QIAGEN, 74104). Library preparation was conducted at BGI, Shenzhen, China, following the guidelines of the standard protocol. Library preparation (BGISEQ-500RS High-throughput sequencing kit, PE50, V3.0, MGI Tech Co, Ltd, Shenzhen, China), hybridization and sequencing were performed according to the manufacturer's standard procedure provided by BGI (BGI-Shenzhen). The sequencing was performed at BGI-Shenzhen using BGISEQ-500. The sequencing data was filtered using SOAPnuke (v1.5.2) software. The processed FASTQ files were mapped to the human transcriptome and genome using HISAT2 (v2.0.4). The genome version was GRCh38, with annotations from Bowtie2 (v2.2.5). Expression level of the gene was calculated by RSEM (v1.2.12) software.

### Statistical analyses

To minimize the phenotypic diversity caused by intra-clonal heterogeneity, which is a common issue for iPSC-related studies, multiple clones from each line were included in the all the analyses. Data was presented as mean ± standard error of the mean (SEM) for the number of samples (n≥3 per clone). Distributions were tested for normality using the Shapiro-Wilk test. Outliers were detected using interquartile range (IQR) and Tukey's Hinges test. The Mann-Whitney U test was used to assess statistical significance for variables with non-normal distribution, while two-sided Student's t-test was applied for normally distributed variables. One-way ANOVA test was used to test the significance among three groups. Data was analyzed with SPSS software (SPSS v.25, IBM) and figures were produced by GraphPad Prism software (Prism 7.0, GraphPad Software, Inc.). Significance was denoted for P values of less than 0.05.

For RNA sequencing analysis, the set of differentially expressed genes identified from pairwise comparisons was identified using DEseq2 (v1.4.5) package (BGI, Wuhan, China). The number of reads per kilobase per million reads (RPKM) method was used to calculate the modification levels of unique genes. Significantly differentially expressed genes were defined as ones with at least 0.3 FPKM level of expression in at least one of the conditions and a q-value less than 0.05 by Bonferroni test. KEGG (https://www.kegg.jp/) enrichment analysis of annotated differentially expressed genes was performed by Phyper based on Hypergeometric test.

## Results

### Generation and characterization of control and POLG patient iPSC-derived astrocytes

We generated POLG patient iPSCs (Table S1) as previously described 30, 31 using fibroblasts of two POLG patients, one homozygous for c.2243G>C; p.W748S (WS5A) and one compound heterozygous c.1399G>A/c.2243G>C; p.A467T/W748S (CP2A). Control iPSCs were generated from two normal human fibroblast lines, Detroit 551 and AG05836B (CTRL). In addition, we employed two ESC lines (HS429 and HS360) as controls 30, 31. To minimize intra-clonal variation, multiple clones for each iPSC line were used, including 4 individual clones for Detroit 551 control line, one clone for AG05836 control and 3 independent clones each for WS5A and CP2A patient lines. A commercial human primary astrocyte line, normal human astrocytes (NHA), was used as control for the astrocyte lineage.

**Figure 1:**
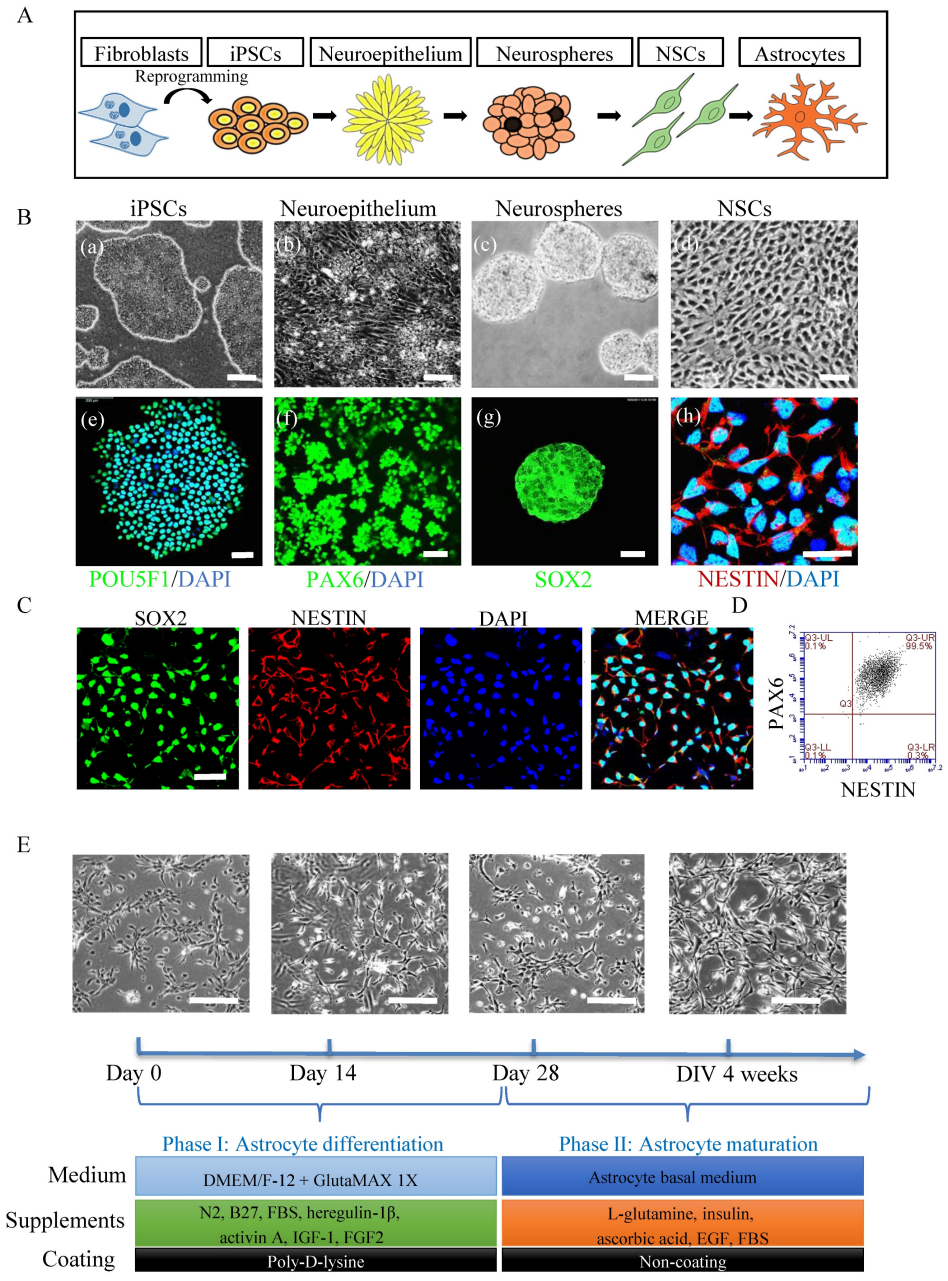
** Generation of astrocytes derived from ESCs, control and POLG iPSCs.** (**A**) Flow chart of reprogramming of iPSCs and astrocytic differentiation via defined stages. (**B**) Representative phase contrast images (upper panel) and immunostaining for specific stages during neural induction from iPSCs to NSCs. Nuclei are stained with DAPI (blue). Scale bar is 50 µm. (**C**) Immunostaining for NSC marker SOX2, NESTIN in iPSC derived NSCs. Nuclei are stained with DAPI (blue). Scale bar is 50 µm (**D**) Representative plots for the percentage of positive staining with neural progenitor markers SOX2 (green) and NESTIN (red) in iPSC derived NSCs. (**E**). Representative phase-contrast images during 4 weeks of astrocyte differentiation from NSCs and cell culture medium information for both differentiation and maturation procedures. Scale bar is 50 µm. The images shown in the figures were obtained from the AG05836B control iPSCs and their derived astrocytes.

To generate astrocytes (Fig. 1A), we first generated NSCs using a previously published protocol 30. Within 5 days of induction, iPSCs (Fig. 1B, (a and e)) underwent morphological changes from a typical compact colony to become a pseudostratified layer of neuroepithelium (Fig. 1B, (b)) that express PAX6 (Fig. 1B, (f)). The PAX6 positive neuroepithelium was then lifted into suspension culture to generate neurospheres (Fig. 1B, (c)) that expressed SOX2 (Fig. 1B, (g)). The resulting spheres were dissociated for further growth into NSC monolayers (Fig. 1B, (d)) which exhibited NESTIN expression (Fig. 1B, (h)). The NSCs expressed the markers SOX2 and NESTIN as evaluated by immunofluorescent staining (Fig. 1C) and showed high purity with over 99% of the cells co-expressing NESTIN and PAX6 as assessed by flow cytometry (Fig. 1D). Next, the iPSC derived NSCs were differentiated to astrocytes (Fig. 1E) using a previously published protocol 33 with some modifications (Table S2). The resulting astrocytes had the typical ramified shape and a stellate morphology (Fig. 1E). After 4 weeks' differentiation, the cells were further matured in maturation medium (Fig. 1E and Table S3) for up to 3 months. All lines described above generated astrocytes with appropriate morphology (Fig. S1).

**Figure 2:**
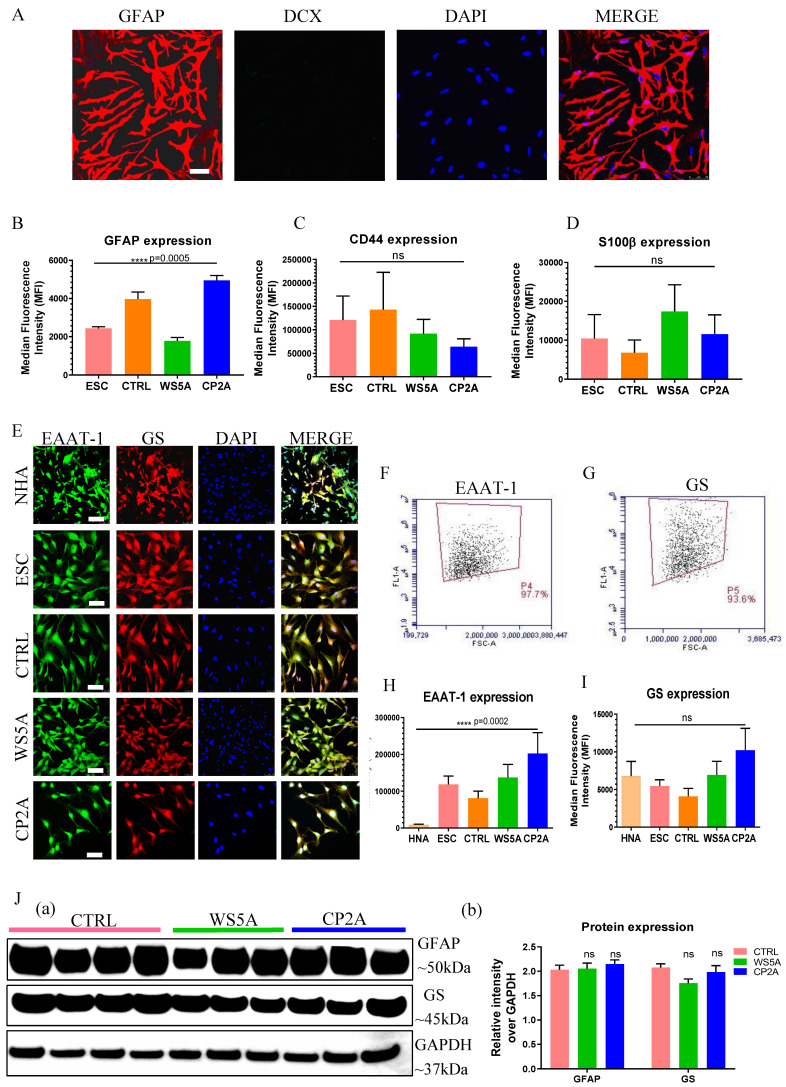
** Characterization of ESC/iPSC-derived astrocytes.** (**A**) Representative confocal images of immunostaining for GFAP (red) and DCX (green) in iPSC-derived astrocytes from AG05836B control. Nuclei are stained with DAPI (blue). Scale bar is 50 µm. (**B-D**) Flow cytometric analysis for protein expression of GFAP (B), CD44 (C) and S100β (D) generated from ESC-derived astrocytes (ESC) and iPSC-derived astrocytes (CTRL, WS5A and CP2A). (**E**) Representative confocal images of immunostaining of NHA, ESC-derived astrocytes (ESC) and iPSC-derived astrocytes (CTRL, WS5A and CP2A) for EAAT-1 (green) and GS (red). Nuclei are stained with DAPI (blue). Scale bar is 50 µm. (**F, G**) Representative plots of flow cytometric quantification by calculating the percentage of positively stained cells EAAT-1 (F) and GS (G). (**H, I**) Flow cytometric measurements for median fluorescence intensity of EAAT-1 (H) and GS (I) in NHA, ESC-derived astrocytes (ESC) and iPSC-derived astrocytes (CTRL, WS5A and CP2A). (**J**) Representative images (a) and expression qualifications (b) in western blot analysis for GFAP and GS in iPSC-derived astrocytes (CTRL, WS5A and CP2A).

We validated the derived astrocytes via ICC and IF staining against key astrocyte markers. We observed expression of the mature astrocyte markers glial fibrillary acidic protein (GFAP) (Fig. 2A and Fig. S2) and S100 calcium-binding protein β (S100β) (Fig. S3). In the commercial NHAs we detected Double Cortin (DCX) expressing cells indicative of neuronal contamination (Fig. S2), but this was not seen in our ESC/iPSC-derived astrocytes (Fig. 2A and Fig. S2). Then the astrocytic identity and purity were assessed by flow cytometric analysis using antibodies against GFAP, CD44 and S100β. All control and patient lines exhibited between 89.4%-99.9% GFAP positive cells, 89.9%-99.8% CD44 positive cells and 80.6%-99.3% S100β positive cells (Fig. S4). While the expression level of GFAP (Fig. 2B), CD44 (Fig. 2C) and S100β (Fig. 2D) varied in different clones, no significant differences were detected in CD44 and S100β and a significant difference was detected in GFAP expression. To examine the functional identity of the astrocytes, we assessed expression of the excitatory amino acid transporter 1 (EAAT-1, also known as GLAST-1) and glutamine synthetase (GS). Immunostaining demonstrated positive expression of both EAAT-1 and GS in NHAs and ESC/iPSC-derived astrocytes (Fig. 2E). Over 90% of the cells showed positive staining for both EAAT-1 (Fig. 2F and S4D) and GS (Fig. 2G and S4E) based on flow cytometric analysis. We found a different level of EAAT-1 expression among different astrocytes (Fig. 2H), but similar expression of GS level (Fig. 2I). Western blot analysis also confirmed that all iPSC derived astrocytes displayed GFAP and GS expression (Fig. 2J (a and b)), with similar expression levels observed in patient astrocytes compared to controls.

This data demonstrates that both patient and control lines can generate robust populations of astrocytes. The above iPSC-derived astrocytes were used for all subsequent experiments to explore disease phenotype and mechanism.

### POLG astrocytes manifest mitochondrial dysfunction and mtDNA depletion

**Figure 3:**
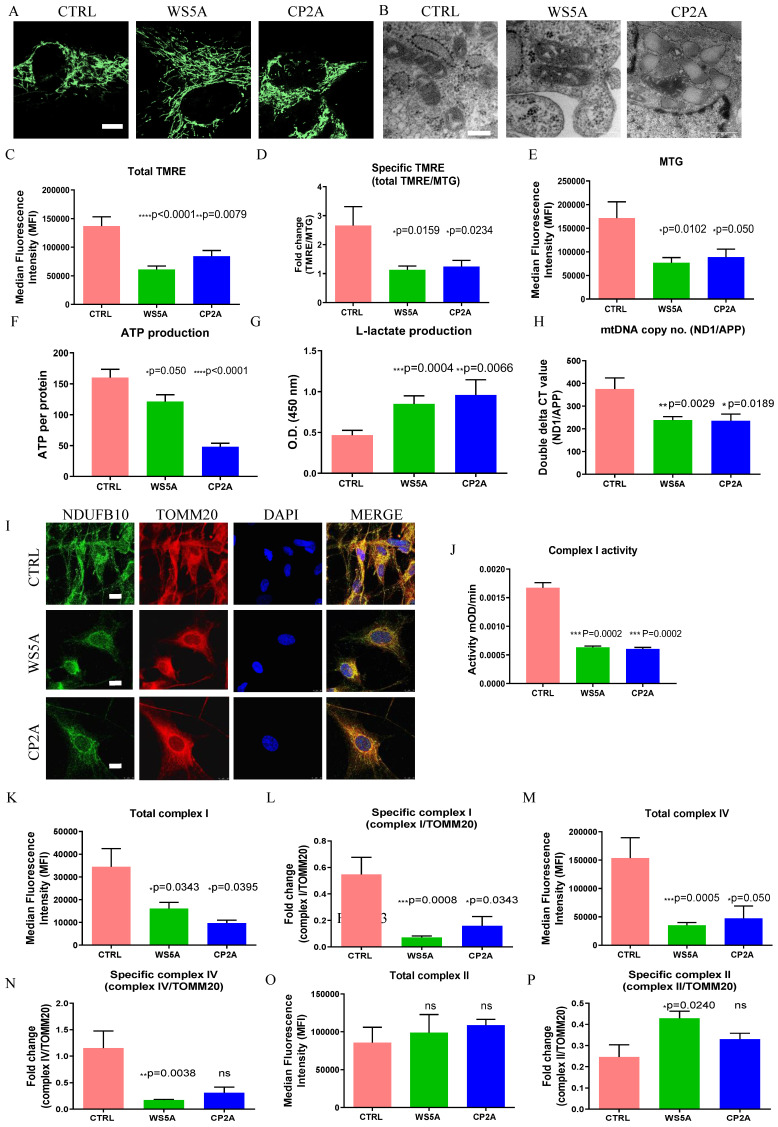
** POLG astrocytes display impaired mitochondrial function, ATP depletion, elevated L-lactate, mtDNA alteration, loss of mitochondrial complex I and IV compared to controls.** (**A**) Representative confocal images of mitochondrial morphology for iPSC-derived astrocytes with staining of MTG. Scale bar is 25 μm. (**B**) Representative transmission electron microscopy images of mitochondrial structures in iPSC-derived astrocytes. Scale bar is 200 nm. (**C, D**) Flow cytometric analysis for MMP at total level measured by TMRE (C) and specific MMP level calculated by total TMRE/MTG (D) in iPSC-derived astrocytes. (**E**) Mitochondrial volume measured by MTG in iPSC-derived astrocytes. (**F**) Measurement of ATP production using LC-MS analysis in iPSC-derived astrocytes. (**G**) Colorimetric analysis for L-lactate generation in iPSC-derived astrocytes. (**H**) Relative mtDNA copy number analyzed by qPCR using primers and fluorogenic probes for regions of mitochondrial* ND1* and nuclear *APP* in iPSC-derived astrocytes. (**I**) Representative confocal images of immunostaining for mitochondrial complex I subunit NDUFB10 (green) and TOMM20 (red) in iPSC-derived astrocytes. Nuclei are stained with DAPI (blue). Scale bar is 10 µm. (**J**) Colorimetric measurements of mitochondrial complex I activities in iPSC-derived astrocytes. (**K-P**) Flow cytometric measurements of mitochondrial complexes, including mitochondrial complex I subunit NDUFB10 at total (**K**) and specific level (**L**), complex IV subunit COX IV at total (**M**) and specific level (**N**), and complex II subunit SDHA protein expression at total level (**O**) and specific level (**P**) in iPSC-derived astrocytes. The specific level was estimated by the ratio of total level over TOMM20.

Next, we assessed mitochondrial parameters in patient and control astrocytes. To visualize mitochondria morphology, we used MTG staining combined with confocal microscopy. A similar mitochondrial morphology was observed in both patient and control astrocytes (Fig. 3A). We also investigated the mitochondrial morphology using transmission electron microscopy and observed similar mitochondrial crista in patient cells compared to control cells (Fig. 3B).

Further, we assessed mitochondrial volume and MMP using MTG and TMRE. Total MMP measured using TMRE alone (Fig. 3C) and specific MMP (Fig. 3D), calculated by the ratio of TMRE/MTG, were both significantly lower in-patient astrocytes than controls. Mitochondrial volume measured using MTG (Fig. 3E) showed significantly lower levels in the patient astrocytes as compared to control cells. Our findings suggest that *POLG* mutations lead to mitochondrial dysfunction in astrocytes with mitochondrial depolarization albeit without major changes in mitochondrial morphology. We then assessed ATP production and L-lactate levels. Using high-performance LC-MS-based metabolomic analysis, we detected a significant drop in ATP production in both WS5A and CP2A astrocytes compared to the controls (Fig. 3F). We also observed significant elevation of L-lactate production in patient astrocytes compared to controls via a colorimetric analysis (Fig. 3G). Next, we investigated alterations in mtDNA copy number using qPCR and found significant mtDNA depletion in POLG patient astrocytes compared to controls (Fig. 3H).

Taken together, our findings confirm that *POLG* mutations have a major impact on iPSC-derived astrocyte mitochondria with lower membrane potential and ATP production, and mtDNA depletion. These changes appear to drive a greater reliance on glucose metabolism for energy metabolism.

### POLG astrocytes display loss of respiratory chain complex I and IV

**Figure 4:**
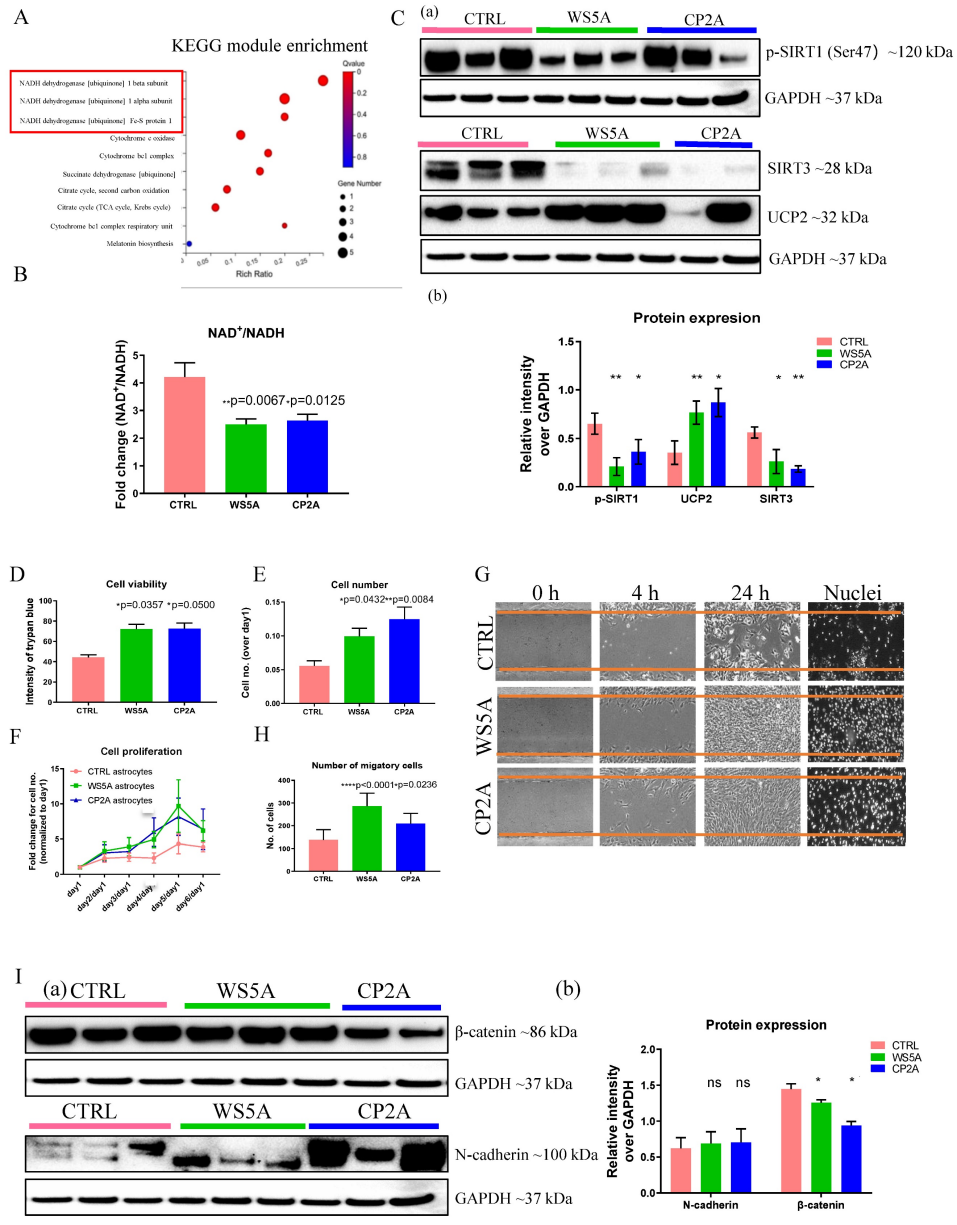
** POLG astrocytes show reactive characteristics with increased cell viability and migratory ability, and display neurotoxicity in 3D spheroids.** (**A**) KEGG module enrichment analysis for downregulated DEGs in POLG astrocytes (WS5A and CP2A) versus control astrocytes (CTRL). (**B**) LC-MS-based metabolomics for quantitative measurement of NAD^+^/NADH ratio in iPSC-derived astrocytes. (**C**) Representative images (a) and quantitation (b) of western blot for p-SIRT1 (Ser47), SIRT3, UCP2 and GAPDH in iPSC-derived astrocytes. The protein expression levels are calculated by normalizing to GAPDH expression. (**D**) Measurement of cell viability using trypan blue staining in iPSC-derived astrocytes. (**E, F**) Cell proliferation assays with quantification of cells numbers on day 5 (E) and growth curve from day 0 to day 6 (**F**) in iPSC-derived astrocytes. (**G, H**). Representative phase-contrast images of wound healing assay for measurement of cell migration at 0, 4 and 24 hours (**G**) and quantification of the migrative cell numbers at 24 hours (**H**) using DAPI staining for iPSC-derived astrocytes. Magnification is 100X. (**I**) Representative images (a) of western blot for β-catenin, N-cadherin and GAPDH and the quantification (b) in iPSC-derived astrocytes.

We showed previously that respiratory chain complex I was lost in POLG affected frontal and cerebellar neurons in micro-dissected post-mortem neurons 15 and we subsequently confirmed this in both iPSC-derived NSCs and DA neurons 30, 31. In agreement with our previous studies, we observed a clear loss of complex I in patient derived astrocytes using immunostaining against NDUFB10 (Fig. 3I). Since the resolution of confocal microscopy is limited, we performed a colorimetric assay, flow cytometry and western blot to confirm this observation. Colorimetric measurement showed decreased complex I enzyme activity in patient astrocytes versus controls (Fig. 3J). Flow quantification of complexes I, II and IV also showed significantly decreased expression of both complex I subunit NDFUB10 (Fig. 4K and L) and complex IV subunit, COX IV (Fig. 3M and N) in patient astrocytes. In contrast, complex II, which is wholly encoded by nuclear DNA, showed similar total expression in both patient lines using assessment of the SDHA subunit (Fig. 3O) and higher specific expression (Fig. 3P) when normalized to TOMM20. Western blot analysis confirmed the lower levels of complex I and IV in patient derived astrocytes compared the controls (Fig. S5), although in WS5A astrocytes this did not reach significance. These data indicate that the respiratory chain defects in astrocytes carrying *POLG* mutations appear more extensive than that seen in post-mortem neurons 34 and NSCs derived from the same iPSCs 30, where only loss of complex I was observed. The expression of voltage-dependent anion channels (VDAC), located in the mitochondrial outer membrane, was similar between patient astrocytes and controls (Fig. S5).

Our data suggest that *POLG* mutations in astrocytes lead to loss of both mitochondrial complex I and IV.

### Transcriptomics confirm lineage identity and metabolic downregulation in POLG astrocytes

We performed RNA sequencing analysis to investigate lineage identity and potential disease-specific changes in gene expression. We compared the transcriptomes of astrocytes derived from three WS5A clones, two CP2A clones and a panel of control lines including NHAs, 2 human ESCs, and the iPSC lines Detroit 551 (3 clones) and AG05836B (1 clone). To compare gene expression between samples, the Pearson correlation coefficients of all gene expression levels between every two samples was calculated, and the correlation coefficient heat maps reflected the overall gene expression between each sample had similar level (Fig. S6A). Lineage identity studies showed significant homogeneity with clustering of astrocytic markers *SOX9*, *NFIX*, *AQP4*, *ALDH1A1*, *GJA1* in all astrocyte samples. Further, we observed no expression of neuronal markers *NEROD1*, *NEUROG2,* and *RELN* or oligodendrocyte markers *OLIG1*, *OLIG2, SOX10* and *NKX2.2* (Fig. S6B).

Next, we investigated if there were any differentially expressed genes (DEGs) between the patient and control lines. We identified 95 genes that were up-regulated and 572 genes that were down-regulated in WS5A astrocytes as compared to controls (Fig. S7A). In CP2A astrocytes, 60 genes were up-regulated and 641 genes down-regulated (Fig. S7B). When comparing CP2A and WS5A astrocyte lines, 15 genes were differentially up-regulated and 24 down-regulated (Fig. S7C). KEGG pathway classifications revealed enrichment for DEGs involved in different pathways including cellular processes, environmental information processing, genetic information process, human disease, metabolism, and organismal systems (Fig. S8A and B). Only glycan biosynthesis and metabolism were enriched when comparing WS5A cells to CP2A group (Fig. S8C).

In our KEGG metabolic pathway analysis, the major enrichment in both WS5A and CP2A astrocytes was for genes related to metabolic pathways. Comparison of patient astrocytes to controls identified the following ten metabolic pathways: (1) Lipid Metabolism, (2) Glycan Biosynthesis and Metabolism, (3) Nucleotide Metabolism, (4) Amino Acid Metabolism, (5) Carbohydrate Metabolism, (6) Metabolism of Other Amino Acids, (7) Metabolism of Cofactors and Vitamins, (8) Xenobiotics Biodegradation and Metabolism, (9) Biosynthesis of the Secondary Metabolite and (10) Energy Metabolism (Fig. S9A and B).

The DEGs in cellular process pathways included 50 genes related to cell growth and death, 43 genes in cellular community (eukaryotes), 15 genes related to cell motility and one gene related to cellular community (prokaryotes) (Fig. S10A). DEGs in the top four metabolic pathways included 188 genes in amino acid metabolism, 154 genes related to glycan biosynthesis and metabolism, 133 genes in nucleotide metabolism and 115 genes in energy metabolism (Fig. S10B). The KEGG pathway enrichment analysis of down-regulated DEGs identified genes that were mainly involved in metabolic pathways and oxidative phosphorylation in both patients compared to controls (Fig. S11A). The correlation network analysis of these pathways showed the highest correlation for metabolic pathways with other related pathways in the network when comparing WS5A (Fig. S12A) and CP2A (Fig. S12B) astrocytes versus controls. The top ten DEGs enriched in KEGG metabolic pathways were *XYLT1*, *PTGS1*, *MGLL*, *ST6GALNAC5*, *NPR*, *HKDC1*, *CSGAL, GALNT15* and *ALDH3A1* in WS5A astrocytes versus controls (Table S4). In CP2A astrocytes, the top ten metabolic pathway DEGs were *ALDH1A1*, *PLEKHA6*, *B4GALNT3*, *ASS1*, *ZBTB7C, GUCY1A1*, *BMPER*, *PDE4B*, and *GCNT3* (Table S5). The enrichment analysis and KEGG pathway module of down-regulated DEGs revealed enrichment for genes involved in NADH dehydrogenase [ubiquinone] 1 beta subunit (Fig. 4A) and the top KEGG module pathway was the NAD biosynthesis aspirate pathway (Fig. S11C) in patient astrocytes lines as compared to controls.

These results confirm that a) our protocol produces astrocytes at high efficiency, b) that these astrocytes are similar to primary human astrocytes in maturity and c) that *POLG* mutations lead to changes in astrocytes through NAD metabolic adaptations.

### POLG astrocytes disturb NAD^+^/NADH via modulating SIRT1/SIRT3/UCP2 pathway

Mitochondrial complex I re-oxidizes NADH to NAD^+^. This vital step is involved in maintaining the NAD^+^/NADH redox balance, mitochondrial metabolism, and ATP production. The redox state also has a major impact on cellular pathways related to neuronal function, plasticity, and brain health 35, 36. We assessed the impact of *POLG* mutations on the absolute levels of NAD^+^ as well as NAD^+^/NADH using LC-MS. As expected, we found a significantly lower NAD^+^/NADH ratio in POLG astrocytes compared to controls (Fig. 4B), however, total NAD^+^ (Fig. S13A) and NADH levels (Fig. S13B) appeared similar in patient and control cell lines.

Sirtuin 1 (SIRT1) and Sirtuin 3 (SIRT3) act as physiological modulators of NAD metabolism 37 and mitochondrial uncoupling protein 2 (UCP2) UCP2 regulates the activity of SIRT3 through sensing the energy level and maintaining mitochondrial homeostasis 38. In light of our findings of altered NAD^+^/NADH levels, we investigated the effect on SIRT1, SIRT3 and UCP2 in POLG astrocytes. We first examined the expression of UCP2, SIRT3 and phosphorylated SIRT1 (Ser47) using western blot analysis and found up-regulation of UCP2 expression and reduced SIRT3 and phosphorylated SIRT1 (p-SIRT1) expression in patient derived astrocytes (Fig. 4C). These data suggest that, in POLG astrocytes, the failure to maintain the NAD^+^/NADH ratio leads to downregulation of the p-SIRT1 and SIRT3 and upregulation of UCP2.

The mitochondrial respiratory chain, particularly complex I, is also considered a major source of ROS. To assess ROS production, we used the cell-permeable probe DCFDA and found a deceased ROS level in patient astrocytes versus control astrocytes (Fig. S14A). To measure the specific ROS level per mitochondrial mass, we calculated the mass via MTDR, then we divided total ROS by mitochondrial mass to give specific ROS and, again, found lower levels in the patient astrocytes, with the difference in WS5A lines reaching significance (Fig. S14B). We then investigated the level of mitochondrial ROS using MitoSox Red and quantified both total and specific ROS as before. We found that the level of total (Fig. S14C) and specific mitochondrial ROS (Fig. S14D) were similar in both patients and controls.

These results suggest that both NAD metabolism and the redox capacity were impaired in POLG astrocytes.

### POLG astrocytes exhibit higher cell proliferation, viability, and migration

Interestingly, during daily culture maintenance, we observed that POLG astrocytes showed an increased growth potential compared to control cells. These observations led us to speculate whether the presence of a *POLG* mutation triggered a shift toward a more aggressive, reactive type of cell, which in turn could contribute to neuronal damage. To answer this question, we investigated cell proliferation, viability, and migration, and analyzed reactive astrocytes markers, includingβ-catenin and N-cadherin. Cell proliferation and viability assays demonstrated significantly increased cell viability (Fig. 4D) and a greater proliferative capacity (Fig. 4E and F) in POLG derived astrocytes compared with controls. We then used a wound-healing assay, monitored by optical microscopy, to assess cell migration. Both visually and after quantification, a greater proportion of patient astrocytes cells crossed the area of injury (gap) when compared with controls, suggesting an enhanced migratory capability in POLG astrocytes (Fig. 4G and H). Western analysis identified higher expression of N-cadherin and a decreased β--catenin expression (Fig. 4I) in *POLG* mutant astrocytes.

Overall, these results suggest that *POLG* mutant astrocytes demonstrated increased growth and migratory abilities, along with altered reactive astrocyte marker expression, suggesting a more aggressive cellular phenotype.

### Both in vivo and in vitro studies reveal reactive astrocytic characteristics in POLG astrocytes

**Figure 5:**
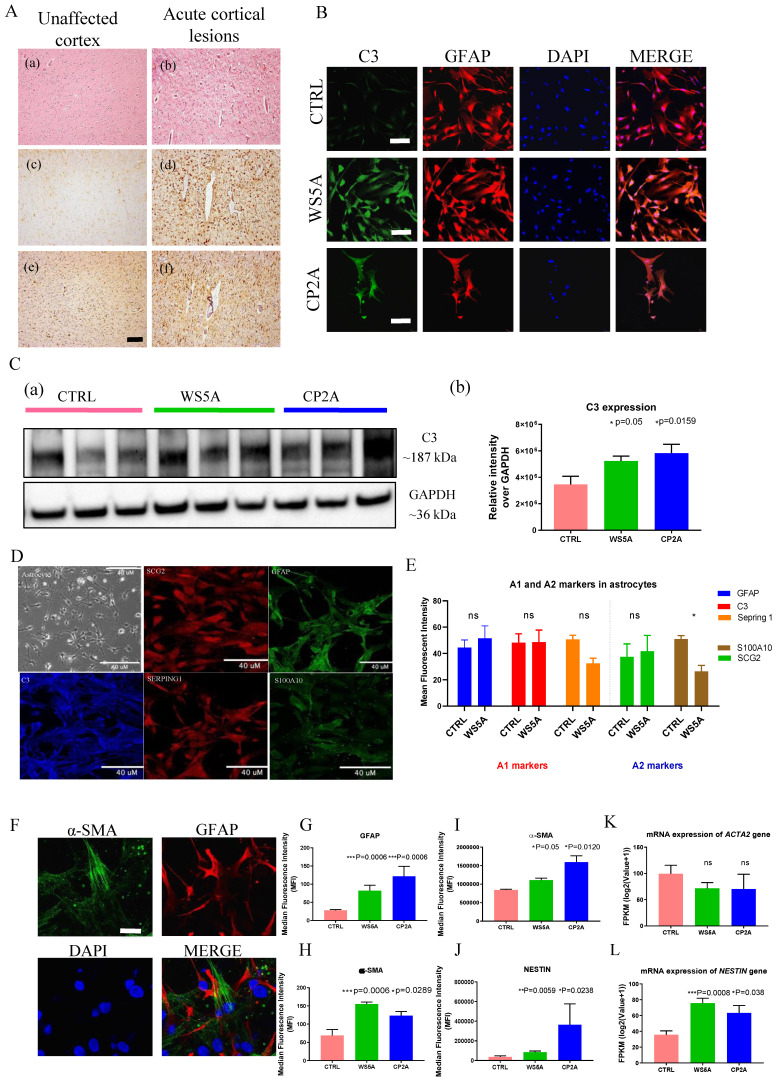
** POLG astrocytes display astrogliosis in brain tissue and express reactive markers in POLG astrocytes compared to controls.** (**A**) Brain histology from a POLG patient with p.W748S homozygous mutation. The left images (a, c, e) show unaffected occipital cortex, whereas the right images (b, d, f) show a cortical stroke-like lesion. H E staining (a and b), GFAP immunohistochemistry (c and d) and HLA DR/DP/DQ immunohistochemistry (e and f) are shown. Scale bar is 50 µm. (**B**) Representative confocal images of immunostaining of control and patient astrocytes for C3 (green) and GFAP (red). Nuclei are stained with DAPI (blue). Scale bar is 50 µm. (**C**) Representative images (a) and quantitation (b) of western blot for C3 and GAPDH in iPSC-derived astrocytes. C3 expression level is calculated by normalizing to GAPDH expression. (**D, E**) Representative confocal images of immunostaining (**D**) and quantification (**E**) of control and patient astrocytes for A1 and A2 markers. Nuclei are stained with DAPI (blue). Scale bar is 50 µm. (**F**) Representative confocal images and quantification of immunostaining of control and patient astrocytes for α-SMA (green) and GFAP (red) in iPSC-derived astrocytes. Nuclei are stained with DAPI (blue). Scale bar is 50 µm. (**G, H**) Quantification of immunostaining for α-SMA (**H**) and GFAP (**G**) from (**F**). (**I, J**) Flow cytometric measurements of α-SMA (**I**) and NESTIN (**J**) in iPSC-derived astrocytes. (**K, L**) RNA sequencing analysis of mRNA expression for *ACTA2* (**K**) and *NESTIN* (**L**) genes in iPSC-derived astrocytes.

Astrogliosis involves the proliferation and migration of astrocytes following neuronal injury 3, 18, 39, 40. This process is induced by numerous pathological stimuli and can contribute to glial scar formation. Previous work 15, 34 showed that astrogliosis commonly accompanies neuronal loss in POLG-disease, both in acute and chronic lesions. To validate this in the context of our current work, we assessed astrocytic and microglial proliferation in the brain of four patients with POLG-disease. Acute cortical lesions (Fig. 5A (b)) showed severe neuronal loss accompanied by pronounced astrogliosis with GFAP accumulation (Fig. 5A (d)) and microglial activation with increased accumulation of HLA-DR/DP/DQ (Fig. 5A (f)). Unaffected cortex showed no visually appreciable changes in the number of astrocytes and/or microglia (Fig. 5A (a), (c) and (e)) while in chronically affected areas of the brain and spinal cord, severe astrogliosis and microgliosis were present (data not shown).

Complement component 3 (C3) is a characteristic and highly up-regulated gene associated with A1 astrocytes 3 and we, therefore, investigated C3 expression in POLG astrocytes. GFAP-positive astrocytes were seen in both patients and control astrocytes (Fig. 5B). We observed an increase toward up-regulation of C3 by western analysis (Fig. 5C). Immunostaining for a range of A1 and A2-like reactive markers revealed a notable increase in the reactive marker GFAP and mild increase of A1-like markers C3 in POLG astrocytes, along with a decrease in the A2-like marker S100A10. However, there were no significant changes observed in the A1-like marker Serping 1 and A2-like marker SCG2 when compared to control cells (Fig. 5D and E). These were performed on three WS5A clones, two CP2A clones and a panel of control lines including 4 clones from the iPSC Detroit 551 and one clone from the AG05836B derived astrocytes.

Evidence from earlier studies using expression profiling suggested that reactive astrocytes express α-SMA and NESTIN 41. We therefore examined these markers in POLG astrocytes. Given that GFAP is the most widely used reactive marker 42, we co-stained with α-SMA and GFAP (Fig. 5F and Fig. S15). Quantification of the fluorescence intensities showed significantly increased expression levels of GFAP (Fig. 5G) and α-SMA (Fig. 5H) in patient astrocytes. Next, we investigated the expression of α-SMA (Fig. 5I) and NESTIN (Fig. 5J) using flow cytometric analysis and found higher expression in patient cells. Analysis of their mRNA levels showed an up-regulation of *NESTIN* (Fig. 5K), but not *ACTA2,* the encoding gene of α-SMA (Fig. 5K).

Gene Ontology (GO) enrichment analyses of up-regulated DEGs revealed significant enrichment of several disease-related processes including response to stimulus, immune system process, cell proliferation, and cell killing (Fig. S16). In the cell-killing pathway, apoptosis was enriched in both WS5A and CP2A astrocytes (Fig. S17A) with *LMNB2* in WS5A and CP2A (Fig. S17B) being the significantly upregulated DEG. Analysis of up-regulated DEGs revealed enrichment for several signaling pathways (Fig. S18), including the Jak-STAT signaling pathway, Wnt signaling pathway and its crosstalk signaling TGF beta pathway, and the PI3K-Akt signaling pathway.

In summary, these studies suggest that POLG induced mitochondrial defects lead to reactive astrocytic characteristics in astrocytes. This phenotype may be regulated by the Jak-STAT, Wntβ-, PI3K-Akt signaling pathways and NADH dehydrogenase [ubiquinone] 1 pathway.

### POLG astrocytes exhibit neurotoxicity

**Figure 6:**
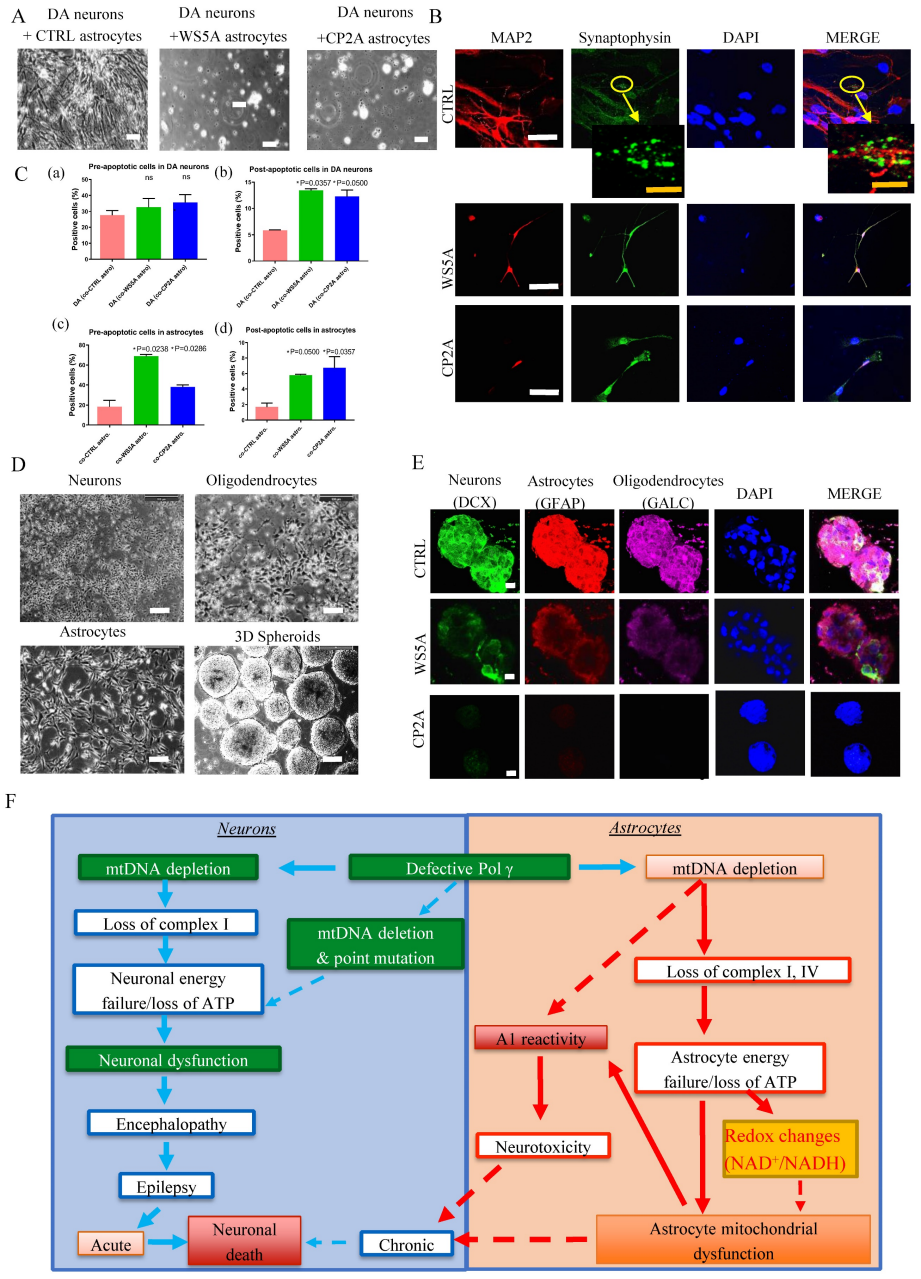
** POLG astrocytes show neurotoxic potential in both direct and indirect neuron/astrocyte co-culture systems.** (**A**) Representative phase-contrast images for DA neurons co-cultured with distinct iPSC-derived astrocytes (CTRL, WS5A and CP2A) for 20 days. Scale bar is 50 µm. (**B**) Representative confocal images for double immunostaining of MAP2 (red) and Synaptophysin (green). The second panel showed the images taken from higher magnification from the cells on the first panel. The yellow arrow shows the synapse signal. Nuclei are stained with DAPI (blue). White scale bar is 10 µm. Orange scale bar is 20 µm. (**C**) Quantification by calculating the percentage of pro-/early and post-/late apoptotic cells in DA neurons co-cultured with astrocytes (a, b) and the percentage of pro-/early and post-/late apoptotic cells in astrocytes co-cultured with DA neurons (c, d). (**D**) Flow chart of the generation of 3D spheroids using hanging drops with combination of iPSC-derived neurons, oligodendrocytes, and astrocytes. Scale bar is 50 µm. (**E**) Representative confocal images of immunostaining for DCX, GFAP and GALC for 3D spheroids composed of neurons, astrocytes, and oligodendrocytes from CTRL, WS5A and CP2A iPSCs. Nuclei are stained with DAPI (blue). Scale bar is 10 µm. (**F**) Summary of the possible disease mechanisms in astrocytes in POLG related disorders.

Next, we asked whether POLG astrocytes could promote neuronal death without cell-cell contact. We generated tyrosine hydroxylase (TH) and tubulin beta III (TuJ1) positive DA neurons (Fig. S19) and astrocytes from both patients and controls. Astrocytes and neurons were grown in separate compartments separated by a membrane that allowed the exchange of small molecular nutrients. After 20 days of co-culture, we assessed the viability of the neurons. We found that control DA neurons died when co-cultured with either WS5A or CP2A reactive astrocytes, but not with control astrocytes (Fig. 6A).

Since astrocytes are involved in synapse formation, we investigated whether *POLG* mutation also impacts this function. To do this, we used the same control DA neurons and performed double immunostaining for the mature neuron marker, MAP2 and the synaptic protein Synaptophysin. We observed small synaptic-like vehicles only in the neurons co-cultured with control astrocytes, but not in those cultured with mutant astrocytes (Fig. 6B). This suggests that POLG astrocytes are either unable to maintain synapses or actively disassemble them.

Given the observed decrease in neuronal viability, we next looked at apoptosis. Using a flow cytometry approach, we investigated Annexin V and PI levels in DA neurons and astrocytes (Fig. S20). After 20 days interaction, we observed increased pre-/early and post-/late apoptotic cell populations in both POLG astrocytes co-cultured with DA neurons and post-/late apoptotic cell populations in DA neurons co-cultured with both POLG astrocytes (Fig. 6C (a), (b) and (d)), whereas the pre- apoptotic DA co-cultured with both POLG astrocytes not reaching significance (Fig. 6C (a)).

We next investigated whether POLG astrocytes were toxic for neurons using a three-dimensional (3D) culture system combining neurons, astrocytes, and oligodendrocytes. Briefly, neurons, astrocytes and oligodendrocytes differentiated from control and patient derived NSCs were counted, and an equal number plated in hanging drops to generate 3D aggregates/spheroids (Fig. 6D). After 20-30 days of co-culture, triple immunofluorescent staining against a neuron marker DCX, an astrocyte marker GFAP, and the oligodendrocyte marker Galactosylceramidase (GALC) was performed. All three cell populations were only seen in control spheroids (Fig. 6E). In patient spheroids, we observed a greatly reduced expression of DCX and GALC, suggesting decreased numbers of neurons and oligodendrocyte (Fig. 6E). This data suggests that *POLG* mutation induces changes in astrocytes that make them toxic for both neurons and mature oligodendrocytes.

Together, these studies suggest that POLG astrocytes with mitochondrial defects and mtDNA depletion can convert to a reactive phenotype that exhibits neurotoxicity.

## Discussion

The role of mitochondria in the pathogenesis of neurodegeneration is an area of intense study. Mutations in *POLG* are the commonest cause of mitochondrial disease and often considered a paradigm for mitochondrial disease in general. *POLG* mutations also drive changes in mtDNA and respiratory chain complex I that mimic what is seen in neurodegenerative disease such as PD and AD making POLG disease an excellent model for studying the mitochondrial component of neurodegeneration. In our study, we investigated how *POLG* mutations, and the mitochondrial dysfunction they induce, affect astrocytes. We found that the abnormal mtDNA homeostasis and complex I deficiency appeared to drive astrocytes into a reactive, toxic state that led to the death of neurons. We dissected the mechanisms involved in this process and showed that these astrocytes manifested increased proliferation, invasion, and upregulation of pathways involved in response to stimulus, immune system process and cell killing.

While we understand that the current state of the art for iPSC studies is to use gene-corrected isogenic controls, the presence of compound mutations, such as we used in this study (CP2A patient), makes the generation of individual controls impracticable. Indeed, it has been stated that the high efficiency of genome cutting, and repair makes the introduction of heterozygous alleles by standard CRISPR/Cas9 technique impossible 43. We chose, therefore, to use healthy, age-matched controls and included both iPSC and ESC lines similar to many other studies, e.g., Chumarina et. al. 44, who used a healthy iPSC control for their iPSC POLG1 studies.

Previous studies, including our own in post-mortem tissue 15, 34 and NSCs 30, have focused on the neuronal consequences of *POLG* mutations. Here, we confirm that iPSC-derived astrocytes also manifest mitochondrial dysfunction. The role of astrocytes in neurodegenerative disease is a rapidly expanding area of interest.

For example, mitochondrial defects and oxidative stress were found in cultured SOD1G93A astrocytes and thought to contribute to motor neuron degeneration in ALS 2. Further, evidence of astrocytic mitochondrial dysfunction was linked to an early event in AD pathogenesis 24. More recently, it was shown that mtDNA depletion induced in astrocytes of mice via the inactivation of Twinkle (TwKO mice) produced chronically activated astrocytes and led to an early onset, spongiotic degeneration of brain parenchyma, microgliosis and secondary neurodegeneration 4. Our findings show that mtDNA depletion caused by defects in the mtDNA polymerase also generates dysfunctional astrocytes that undergo reactive transition. Our *in vitro* system also allows us to demonstrate that these astrocytes are indeed toxic for neurons suggesting that they are actively involved in the disease process.

Loss of respiratory chain complex appears to play a crucial role in neurodegeneration 45. Whether this loss is a primary or secondary event is, however, currently unresolved. In PD, complex I loss was shown to affect the whole brain 46, not just the substantia nigra 46, 47, suggesting that it may not be the primary cause of neuronal death, but a secondary consequence. We also saw loss of cytochrome c oxidase (COX), complex IV, in POLG astrocytes. This is different to what we found in NSCs, and post-mortem tissue 15. With regard to the latter, COX staining in tissues identifies the largest cells, i.e., in this case neurons. It is possible, therefore, that any defect in astrocytes would be lost against the background of neuronal staining. Since *POLG* mutations cause mtDNA copy number loss, it is clearly possible that both complexes are lost due to mtDNA depletion. Interestingly, accumulating evidence suggests that complex IV stabilizes the assembly of complex I and that inhibition of complex IV expression can impair complex I assembly in mouse cell lines 48.

In POLG diseases, we and others 15, 49, 50 have demonstrated a complex I defect in tissues, and we have confirmed this in iPSC-derived NSCs and neurons 30, 31. The loss of complex I is associated with failure to maintain the redox state of the cell, shown by a decreased NAD^+^/NADH ratio, suggesting that in POLG disease, complex I is a primary or at least an important mechanistic event. In addition to the altered redox state, we see impaired mitochondrial energy metabolism shown by lowered ATP production. Emerging evidence implicates NAD^+^ depletion in a wide range of age-related diseases and neurodegenerative diseases 36, 51, 52. This present study confirms the link between complex I deficiency and altered NAD^+^/NADH ratio and shows that this also occurs in astrocytes.

Under normal conditions, astrocytes are the most prevalent cell type in the brain and perform supportive roles for neurons and neuronal circuits. Damage to the central nervous system (CNS) induces the formation of reactive astrocytes that proliferate and generate glial scarring (gliosis) that surrounds the lesion and separates it from adjacent neural tissue 1, 53-55. Given the clear complex I deficiency and abnormal redox ratio, we postulated that NAD^+^ repletion could improve mitochondrial dysfunction and thus reverse the reactivity seen in POLG astrocytes. Our results illustrate that *POLG* mutated astrocytes lose their ability to support neuronal growth and instead lead to neural death. These astrocytes show increased cell viability, greater proliferative capacity, and greater ability to migrate, with up-regulation of calcium-dependent N-cadherin.

POLG “reactive-like” astrocytes appear able to trigger toxicity in neurons in two different ways: first, via direct neuron-astrocyte communication and secondly in a cell autonomous fashion. Direct toxicity may be mediated by N-cadherin up-regulation. Similar to our findings, a previous study demonstrated that Ca^2+^-dependent translational regulation of N-cadherin expression in astrocytes was involved in reactive astrogliosis following brain injury 56. Cell autonomous astrocyte-dependent toxicity must act via secreted factors: this may include factors such as the up-regulation of pro-inflammatory factors and/or the downregulation of synaptogenic factors. Further investigation is necessary to define whether astrocytic reactivity is a primary pathological feature of POLG related diseases, and to identify the humeral factors released by astrocyte. Nevertheless, a previous study in mice with astrocyte-specific mtDNA depletion clearly demonstrated that these cells were sensitive to changes in mitochondrial function and that loss of mtDNA could lead to neuronal loss 4.

Activated astrocytes show hypertrophy and express a stereotypical array of cytoskeletal proteins, most prominently GFAP. Previous reports 3 showed that induction of a subtype of inflammation-associated, reactive astrocytes, that promote death of both neurons and oligodendrocytes, was mediated by activated microglia and others have suggested that this occurs in various neurological diseases 5, 17, 29. In the present study, we show that POLG astrocytes *in vitro* exhibit uniform polarization into LPS-induced neuroinflammation A1 subtype nor ischemia-induced A2-reactive states, and display a mixed phenotype characterized by the up-regulation of GFAP and α-SMA/NESTIN, two cytoskeletal proteins expressed during astrocyte development. In agreement with our results, reactive astrocytes isolated from MS lesions show significant up-regulation of α-SMA and NESTIN markers 41. In addition, those studies also reveal the regulatory role of TGF‐β1 in the organization and expression of these cytoskeletal proteins.

Building on our previous research 31, 32, our study proposes a model that hypothesizes the pathogenic changes in POLG-related disorders, differentiating between the effects on neurons and astrocytes (Fig. 6F). In neurons, the proposed sequence of events begins with mtDNA depletion, which results in the loss of complex I activity. This initiates a cascade of neuronal energy failure due to ATP depletion, resulting in neuronal dysfunction. The dysfunction manifests clinically as encephalopathy and epilepsy, which can progress to acute neuronal death or contribute to chronic neurodegeneration. In astrocytes, mtDNA depletion leads to the loss of complexes I and IV. This parallels a similar energy failure as seen in neurons, marked by ATP loss. The energy failure in astrocytes is coupled with A1 reactivity and redox changes, specifically alterations in the NAD^+^/NADH ratio, contributing to astrocyte mitochondrial dysfunction. The dysregulated astrocytes add to neuronal damage through neurotoxicity, potentially exacerbating the neuronal decline. Both paths suggest that defective POLG leads to significant mitochondrial dysfunction in neurons and astrocytes, underpinning the neurological symptoms observed in POLG-related diseases. The dashed lines indicate a feedback loop where mitochondrial dysfunction in astrocytes can further influence neuronal viability, creating a vicious cycle of cellular damage.

Our study provides a comprehensive analysis of the KEGG pathways disrupted in POLG-related disorders, unraveling the intricacies of mitochondrial dysfunction and its downstream effects on astrocyte metabolism and neuron-astrocyte interactions. The implications of these disrupted pathways extend beyond mere energy production deficits, offering insights into the pathophysiological processes underpinning neurodegeneration in POLG diseases. Mitochondrial dysfunction is the hallmark of *POLG* mutations, as evidenced by the perturbations in pathways related to the TCA cycle and oxidative phosphorylation. This mitochondrial compromise leads to an energy crisis within astrocytes and neurons, further exacerbating the pathogenic cascade. The loss of membrane potential and the reduction of ATP production are not just byproducts of impaired mitochondria but are also potential instigators of the neurodegenerative process. The alterations in lipid and amino acid metabolism pathways have significant implications for neuronal integrity and function. Lipids are crucial for maintaining cellular membranes and signaling, while amino acids serve as precursors for neurotransmitters and are vital for maintaining synaptic function. Our findings suggest that the perturbations in these pathways may contribute to altered membrane fluidity, disrupted synaptic function, and impaired neurotransmitter synthesis, culminating in the neurological manifestations seen in POLG-related diseases. Interestingly, our data indicate an astrocytic shift towards a phenotype characterized by enhanced proliferation and migration, aligning with the observed astrogliosis in patient samples. This phenomenon may not only represent a glial scar formation response but also points to a more pervasive role of astrocytes in disease propagation, wherein they could actively contribute to the neurotoxic milieu. The presence of neuroinflammatory markers among the KEGG pathways underscores the potential role of chronic inflammation in disease progression. Inflammation is a double-edged sword; while initially protective, chronic neuroinflammation can lead to a deleterious cycle of neuronal damage. Our findings elucidate the contribution of inflammatory pathways, suggesting that *POLG* mutations might instigate or amplify the neuroinflammatory response, thus playing a pivotal role in disease pathogenesis. Synaptic dysfunction is a critical element of cognitive decline in neurodegenerative diseases. The downregulation of pathways governing synaptic structure and function may result in compromised neuronal communication and plasticity. The resulting synaptic deficits could underline the cognitive and motor dysfunctions characteristic of POLG diseases. The nucleotide metabolism pathways resonate with the fundamental DNA repair and replication defects in POLG-related disorders. The inability to maintain mtDNA integrity could lead to a cascade of cellular events culminating in cell death, highlighting the vulnerability of postmitotic cells such as neurons to genomic instability. The dysregulation of signaling pathways such as Jak-STAT, Wnt, and TGF-beta could provide a molecular basis for altered cell communication, differentiation, and response to external stimuli. These pathways are integral to cellular homeostasis and the disruption of such signaling could have far-reaching implications on cell fate and function. In summary, our exploration into the KEGG pathways associated with *POLG* mutations has shed light on the broad cellular disturbances that culminate in neurodegeneration. These findings underscore the complexity of POLG diseases and the importance of astrocytes in their pathogenesis. By delineating the metabolic and signaling derangements, we not only enhance our understanding of the disease mechanism but also open avenues for potential therapeutic interventions targeting specific metabolic and inflammatory pathways.

## Conclusion

Our current studies demonstrate that *POLG* mutations induce similar mitochondrial changes in astrocytes as are found in POLG neuronal progenitors and post-mortem neurons 30, 31, namely mtDNA depletion, loss of membrane potential and complex I and redox changes. In addition, our current studies provide compelling evidence for the involvement of astrocytes in the disease process initiated by the presence of *POLG* mutations. This allows us to hypothesize that neuronal death in POLG related disease may occur by two mechanisms. Firstly, processes driven by changes in the neurons themselves: loss of ATP with changes in redox potential and ROS production can lead to neuronal dysfunction and chronic neuronal loss. The initiation of seizures in already stressed neurons will exceed the capacity of the neuron to maintain energy metabolism leading to loss of cellular integrity and acute neuronal death. The second, novel mechanism is based on our current work and involves astrocytic toxicity: metabolic changes in astrocytes lead to reactivity and toxicity and eventually to chronic neuronal loss (Fig. 6F). Lastly, we believe our model system offers great tractability both for investigating mechanisms involved in human disease initiation and progression, and as therapeutic targets, not just for POLG related diseases, but also in the broader context of neurodegeneration in which mitochondrial defect occurs.

## Supplementary Material

Supplementary figures and tables.

Supplementary Key Resources Table.
