# Supplementary material for: Activation of Neurotoxic Astrocytes Due to Mitochondrial Dysfunction Triggered by POLG Mutation
Source: Int J Biol Sci. 2024 May 11;20(8):2860–80. doi: 10.7150/ijbs.93445 (PMC11186360; doi:10.7150/ijbs.93445)
Supplement: Supplementary file 1 — Supplementary figures and tables. [file ijbsv20p2860s1.pdf]

Table S1: Summary of the information for the cells used in this study.

Lines	Status	Sex	Age at Biopsy	Mutation
NHA	control	F	Fetal	no
ESC1 (HS429)	control	F	Embro / 6 days	no
ESC2 (HS360)	control	M	Embro / 6 days	no
Detroit 551	control	F	Fetal	no
AG05836B	control	F	44 yrs	no
WS5A	POLG disease	F	44 yrs	c.2243G>C; p.W748S
CP2A	POLG disease	M	49 yrs	c. 1399G>A; p.A467T and c.2243G>C; p.W748S

Table S2. Components of the astrocyte differentiation medium.

**Medium/Supplements**	**Product no.**	**Concentration**
DMEM/F-12 + GlutaMAX	Gibco, cat. no. 10565-018	500 mL
N2-100x	Gibco, cat. no. 17502-048	5 mL (1x)
B27-50x	Gibco, cat. no. 17504-044	10 mL (1x)
fibroblast growth factor-2	Peprotech, cat. no. 100-18B	4 µg (8 ng/mL)
activin A	Peprotech, cat. no. 120-14E	5 µg (10 ng/mL)
heregulin-1β	Sigma-Aldrich, cat. no. SRP3055	5 µg (10 ng/mL)
insulin-like growth factor-1	Sigma-Aldrich, cat. no. I3769	100 µg (200 ng/mL)
foetal bovine serum	Sigma-Aldrich, cat. no. 12103C	5 mL (1%)

Table S3. Components of the astrocyte maturation medium.

Medium/Supplements	Product no.	Concentration
Astrocyte Basal Medium	Lonza, cat. no. CC-3187	500 mL
gentamicin	Lonza, cat. no. 17-518Z	25 mg (50 ng/mL)
epidermal growth factor	Gibco, cat. no. PHG0314	10 µg (20 ng/mL)
ascorbic acid	Sigma-Aldrich, cat. no. A4034	500 µg (1 µg/mL)
FBS	Sigma-Aldrich, cat. no. 12103C	15 mL (3%)
L-glutamine	Sigma-Aldrich, cat. no. G7513	5 mL (1%)
insulin	Roche, cat. no. 11376497001	1.25 mL (0.25%)

Table S4. List of the top 10 DEGs in KEGG metabolism pathway in WS5A astrocytes versus control astrocytes.

**Gene ID**	**Gene Symbol**	**Qvalue (WS5A-vs-CTRL)**
64131	*XYLT1*	1.24E-07
5742	*PTGS1*	5.31E-05
11343	*MGLL*	6.62E-04
81849	*ST6GALNAC5*	9.31E-04
4881	*NPR1*	0.001204153
80201	*HKDC1*	0.001337
55790	*CSGAL*	0.00140215
117248	*GALNT15*	0.002062158
218	*ALDH3A1*	0.002867196

Table S5. List of the top 10 DEGs in KEGG metabolic pathway in CP2A astrocytes versus control astrocytes.

Gene ID	Gene Symbol	Qvalue (CP2A-vs-CTRL)
216	*ALDH1A1*	4.16E-11
22874	*PLEKHA6*	1.67E-06
283358	*B4GALNT3*	1.47E-05
445	*ASS1*	1.78E-05
201501	*ZBTB7C*	1.80E-05
2982	*GUCY1A1*	4.94E-05
168667	*BMPER*	1.58E-04
5142	*PDE4B*	2.05E-04
9245	*GCNT3*	2.23E-04

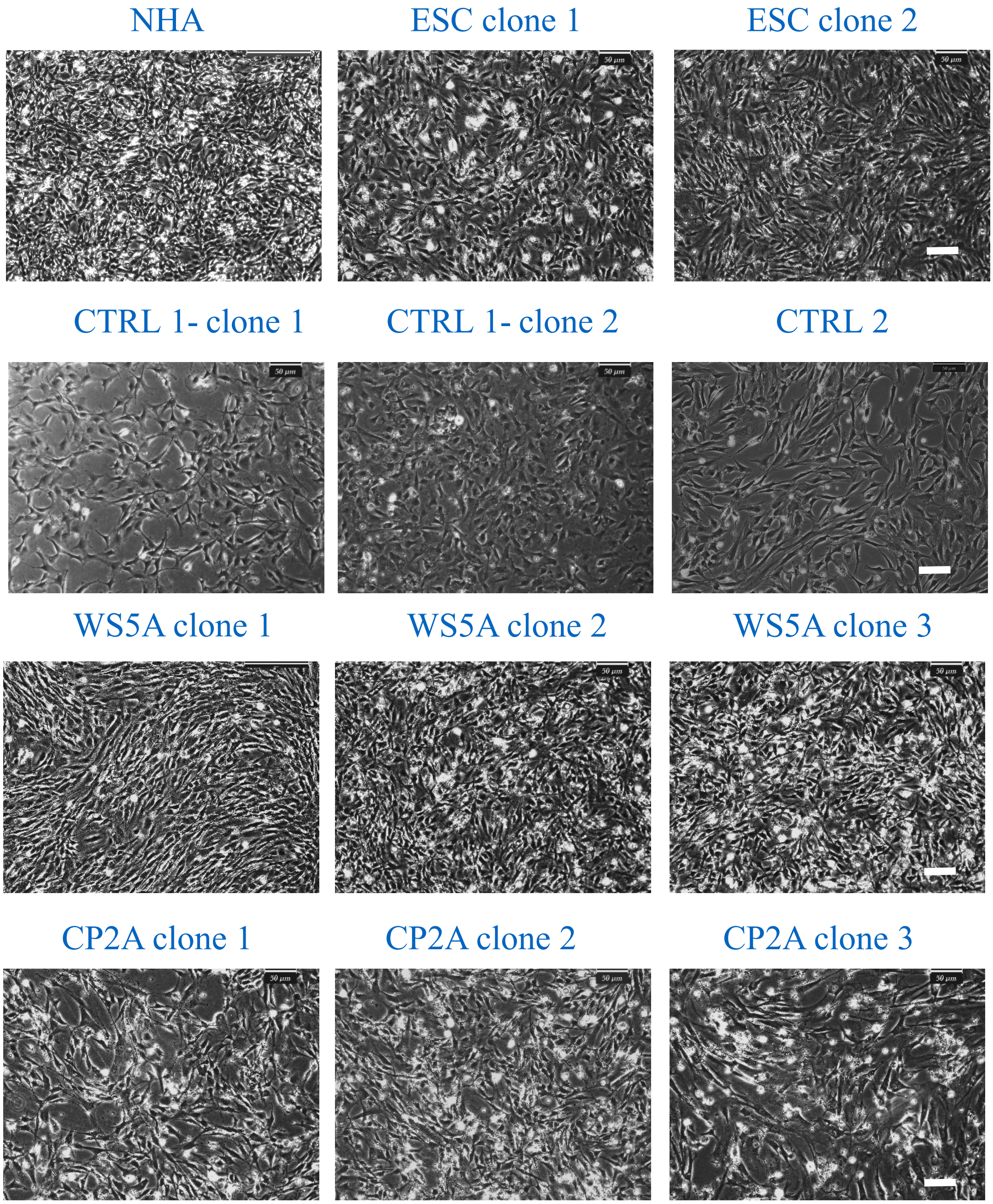

Figure S1. Representative phase-contrast images of NHA, ESC-derived astrocytes and iPSC-derived astrocytes from control and patients carrying homozygous and heterozygous *POLG* mutations (WS5A and CP2A). Related to Figure 1 and 2. Scale bar is 50 µm.

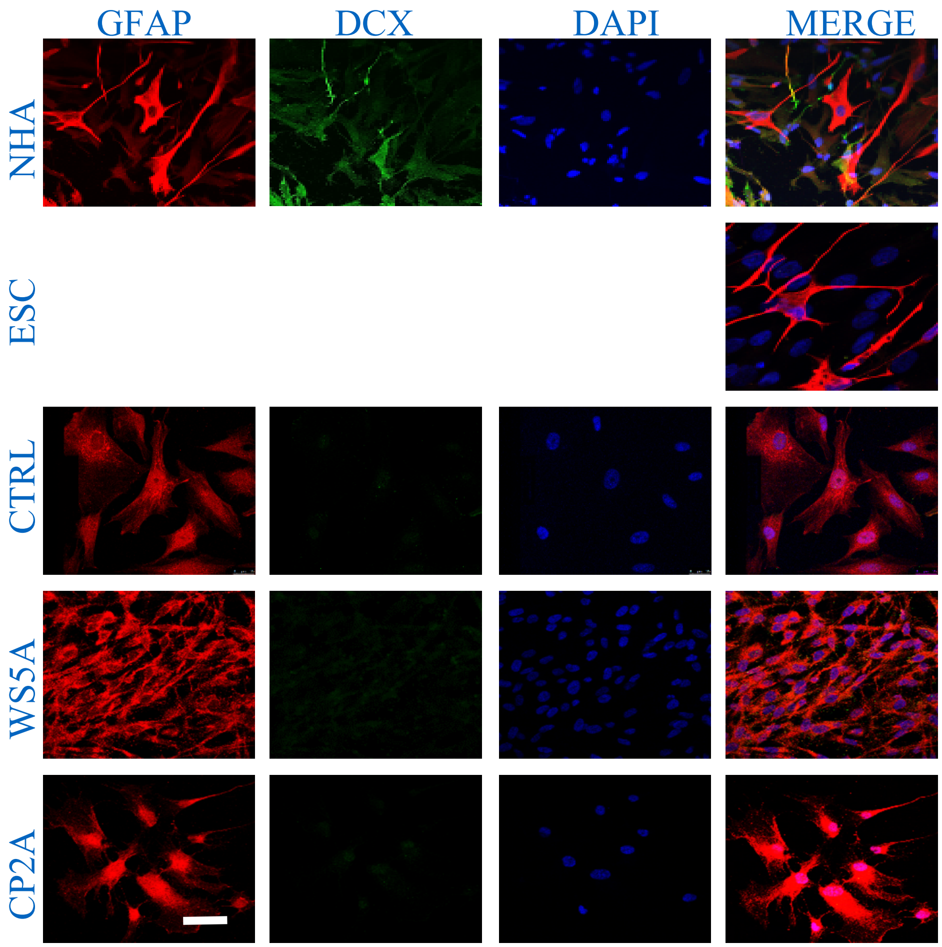

Figure S2. Representative confocal images of immunostaining for GFAP (red) and DCX (green) in NHA and ESC-derived astrocytes and iPSC-derived astrocytes from control and patients carrying homozygous and heterozygous *POLG* mutations (WS5A and CP2A). Nuclei are stained with DAPI (blue), related to Figure 1 and 2. Scale bar is 50 µm.

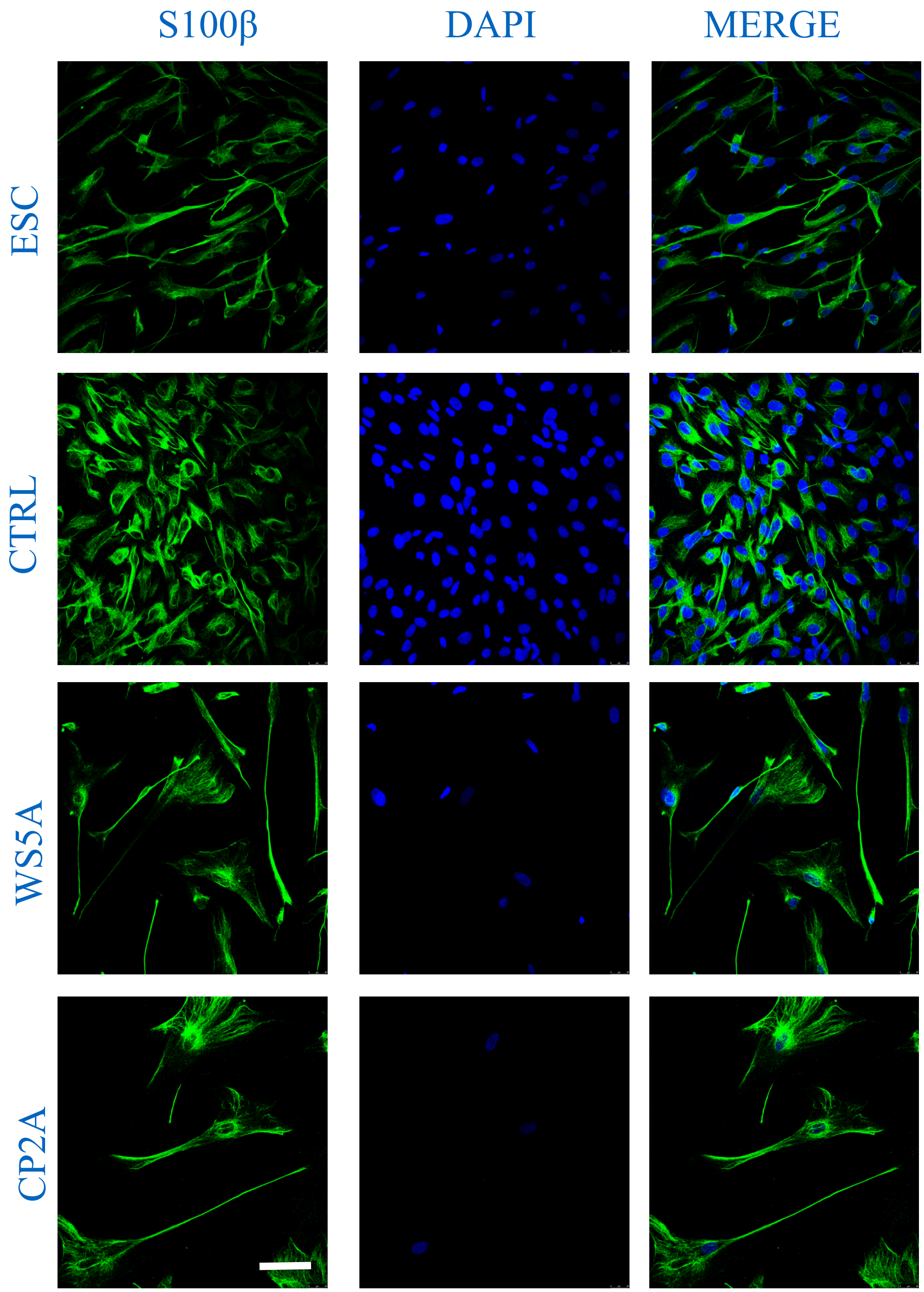

Figure S3. Representative confocal images of immunostaining for S100β (green) in ESC-derived astrocytes and iPSC-derived astrocytes from control and patients carrying homozygous and heterozygous *POLG* mutations (WS5A and CP2A). Nuclei are stained with DAPI (blue), related to Figure 1 and 2. Scale bar is 50 µm.

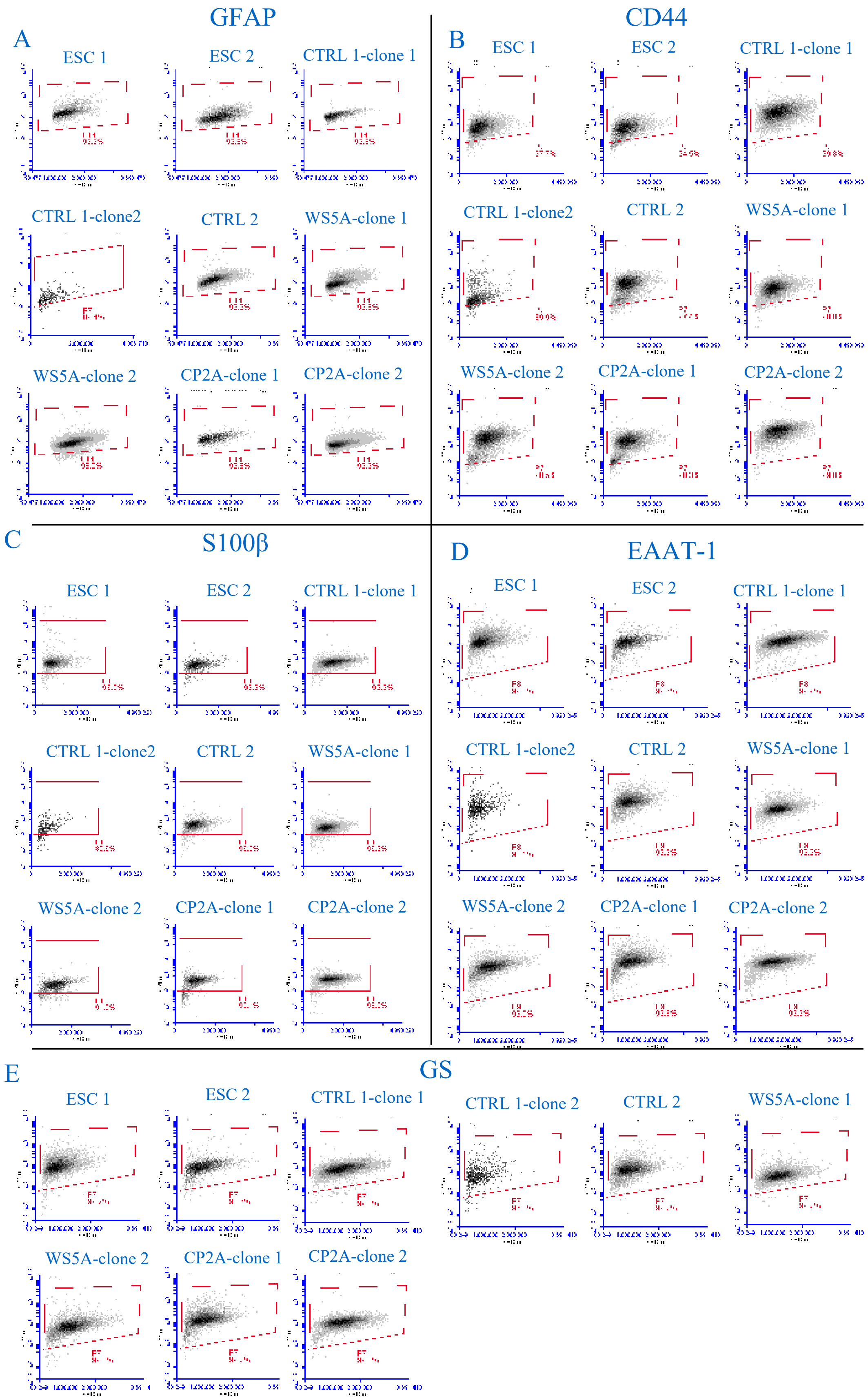

Figure S4. Representative plots from flow cytometric analysis of the positive cell population stained with astrocyte markers GFAP (A), CD44 (B), S100β (C), EAAT1 (D) and GS (E) in astrocytes, related to Figure 1 and 2.

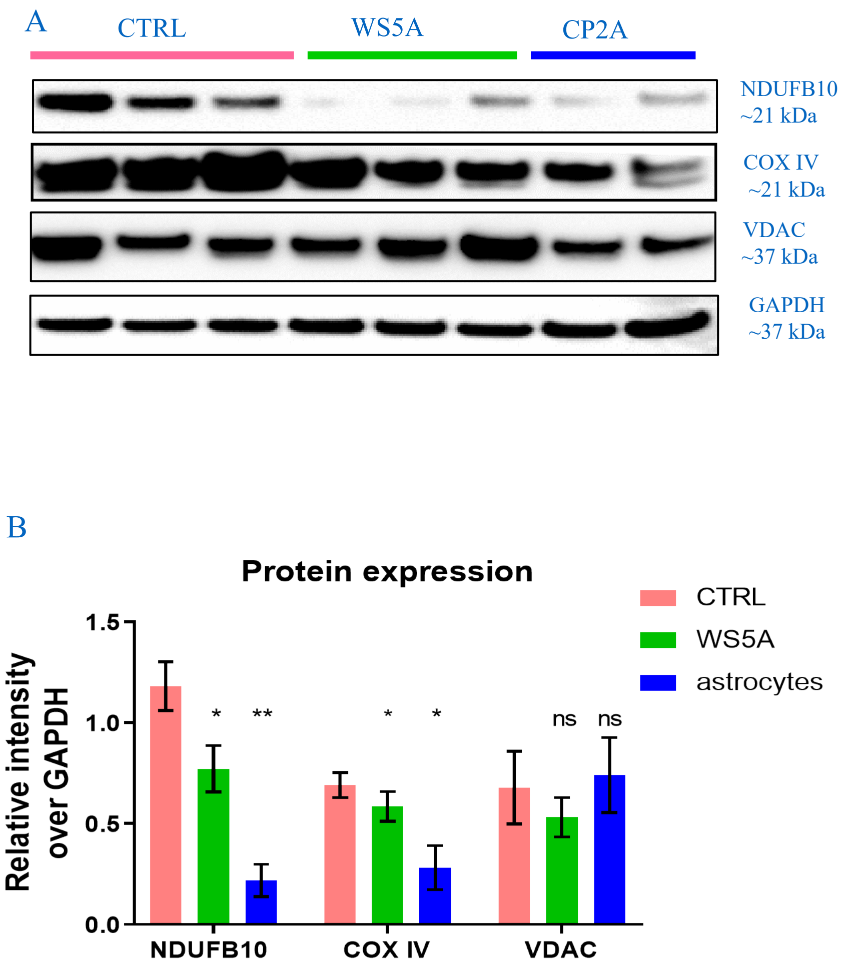

Figure S5. Representative images (A) and quantitation of the protein expression (B) of western blot for mitochondrial complex I (NDUFB10), complex IV (COX IV), VDAC and GAPDH.

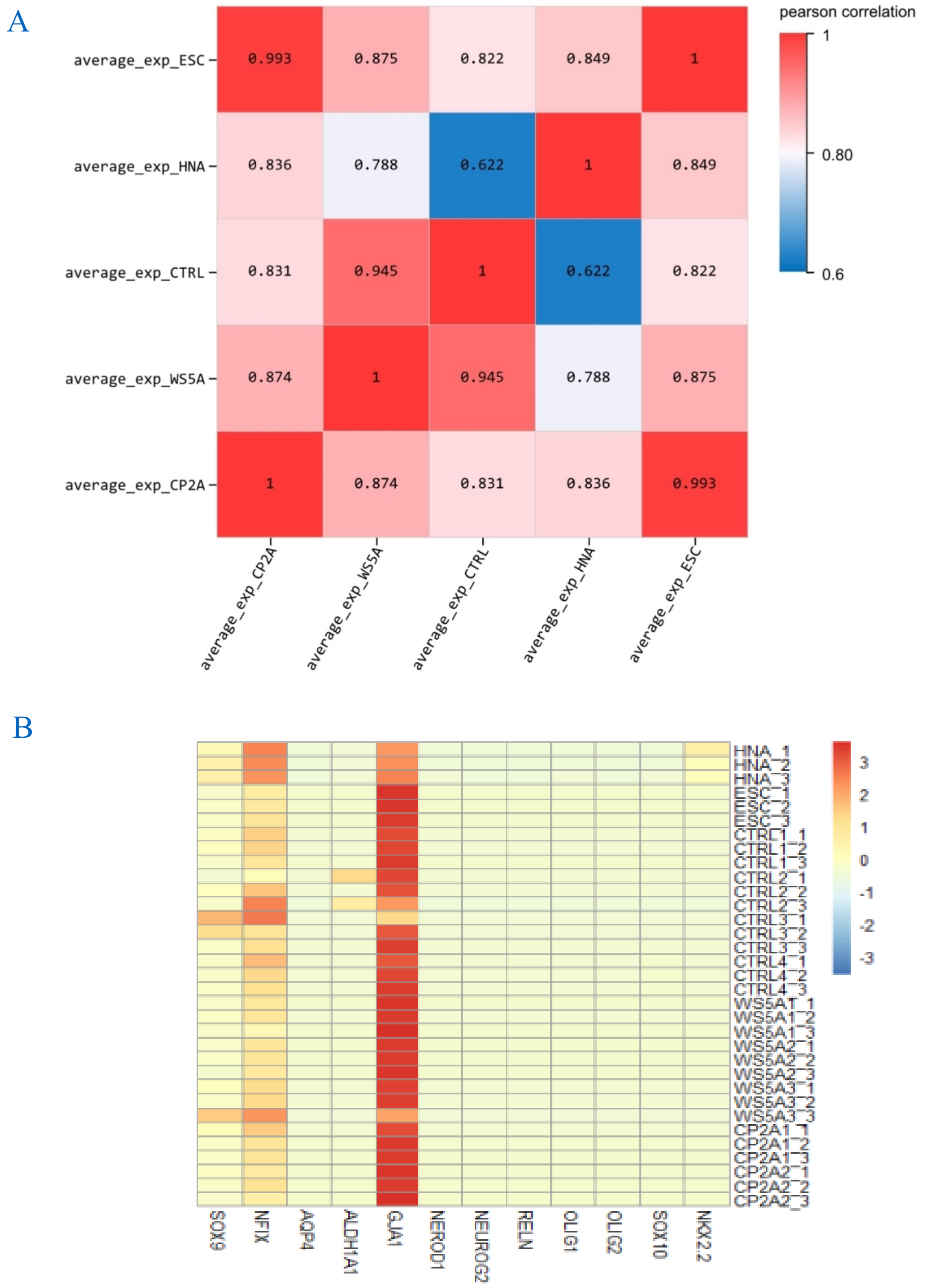

Figure S6. RNA sequencing analysis shows a transcriptomic profile similar to primary astrocytes and between two patient lines, and the similar astrocyte lineage identity in iPSC-derived astrocytes compared to NHA. (**A**) Correlation heat map of gene expression profiles in individual groups. (**B**) Gene expression of astrocyte, neuron, and oligodendrocyte markers from NHA and ESC/iPSC-derived astrocytes. The different markers are listed on the x-axis, and cell type and donors on the y-axis. Results are expressed as Fragments Per Kilobase Million (FPKM).

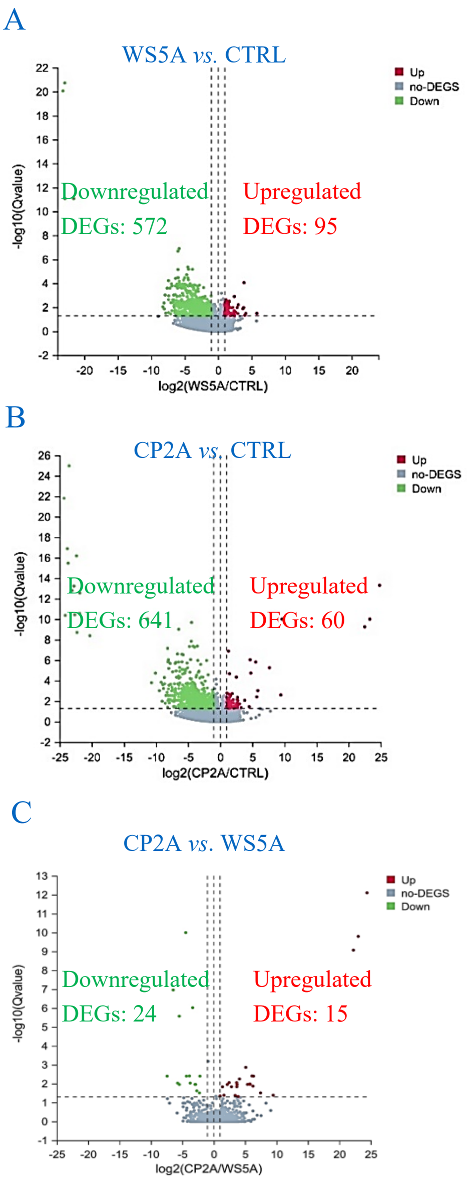

Figure S7. Transcriptomic profiling shows the similar astrocyte lineage identity in iPSC-derived astrocytes compared to NHA and different profiling in POLG astrocytes compared to control. (**A -C**) Scatter plots for DEGs in POLG astrocytes compared to control astrocytes. Each point represents an identified gene and the log2 difference intensity is plotted against the t-test p value for each gene. Genes with significantly ($P<0.05$) upregulated levels between the two samples are in red while downregulated genes are in blue.

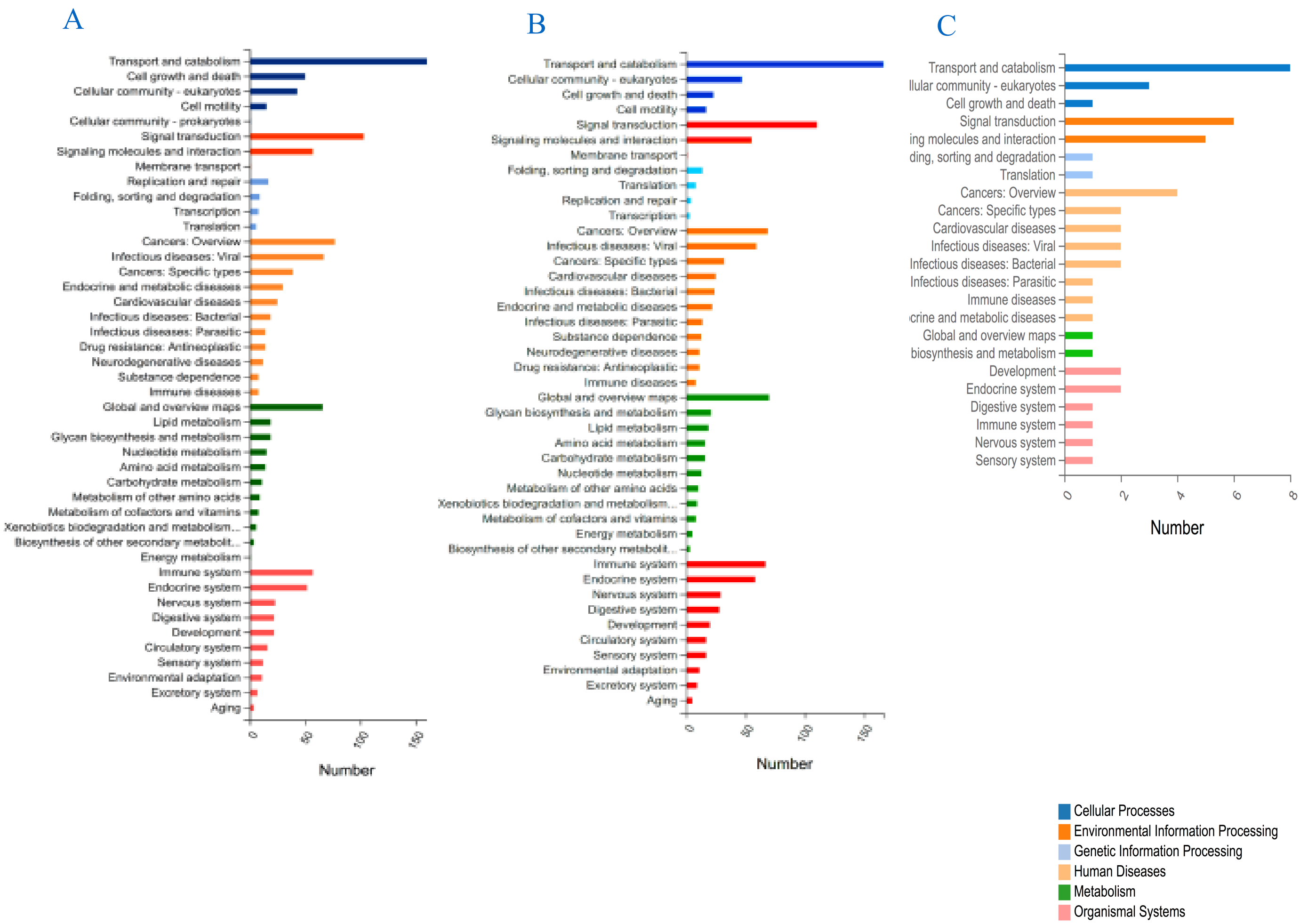

Figure S8. KEGG pathway classification for DEGs pathway in WS5A (A) and CP2A (B) astrocytes versus control astrocytes and WS5A versus CP2A astrocytes (C).

A

B

Figure S9. KEGG metabolic pathway analysis for down-regulated DEGs in WS5A astrocytes compared to control group (A) and CP2A astrocytes compared to control group (B).

A

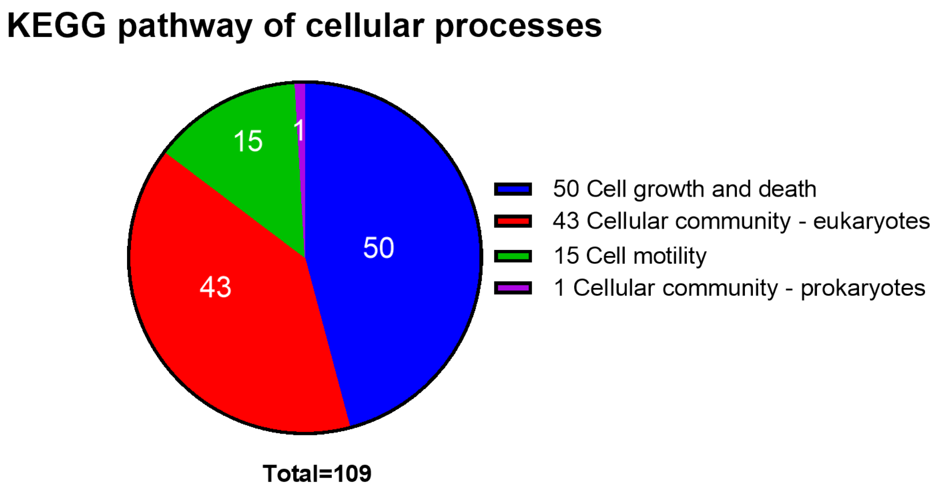

B

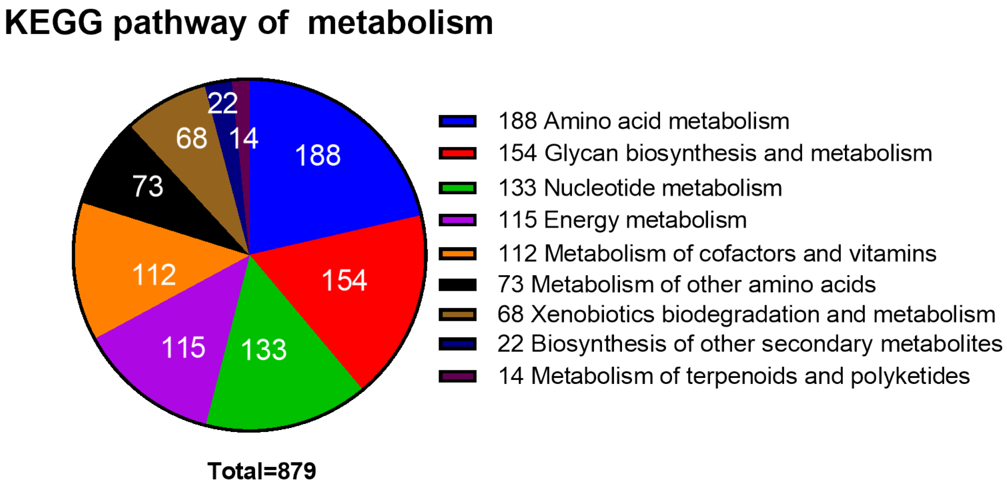

Figure S10. KEGG pathway analysis and cellular processes (A) and metabolism (B) in POLG astrocytes versus control astrocytes.

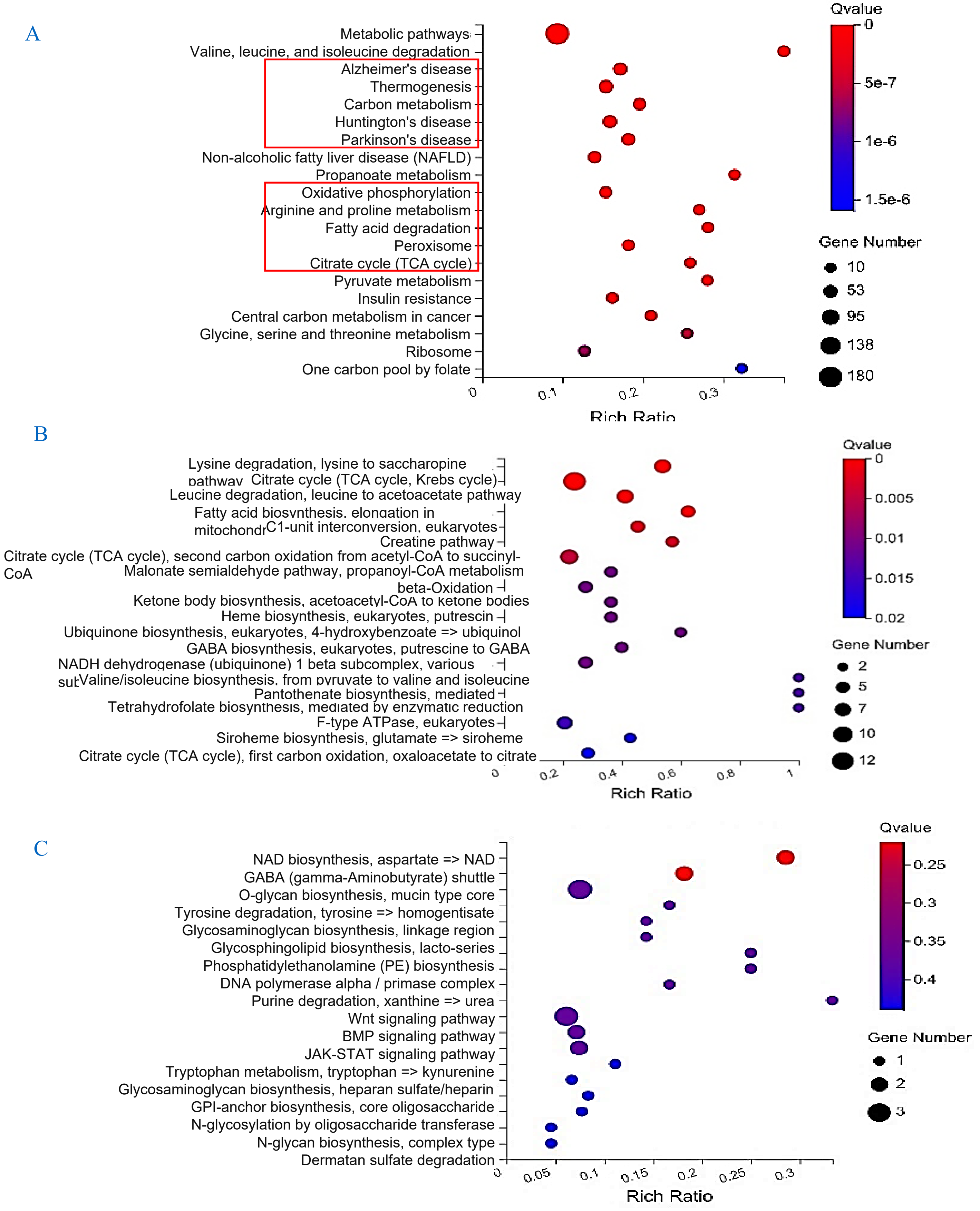

Figure S11. KEGG pathway enrichment analysis (A) and KEGG module enrichment analysis for DEGs (B) and downregulated DEGs (C) in POLG astrocytes versus control astrocytes.

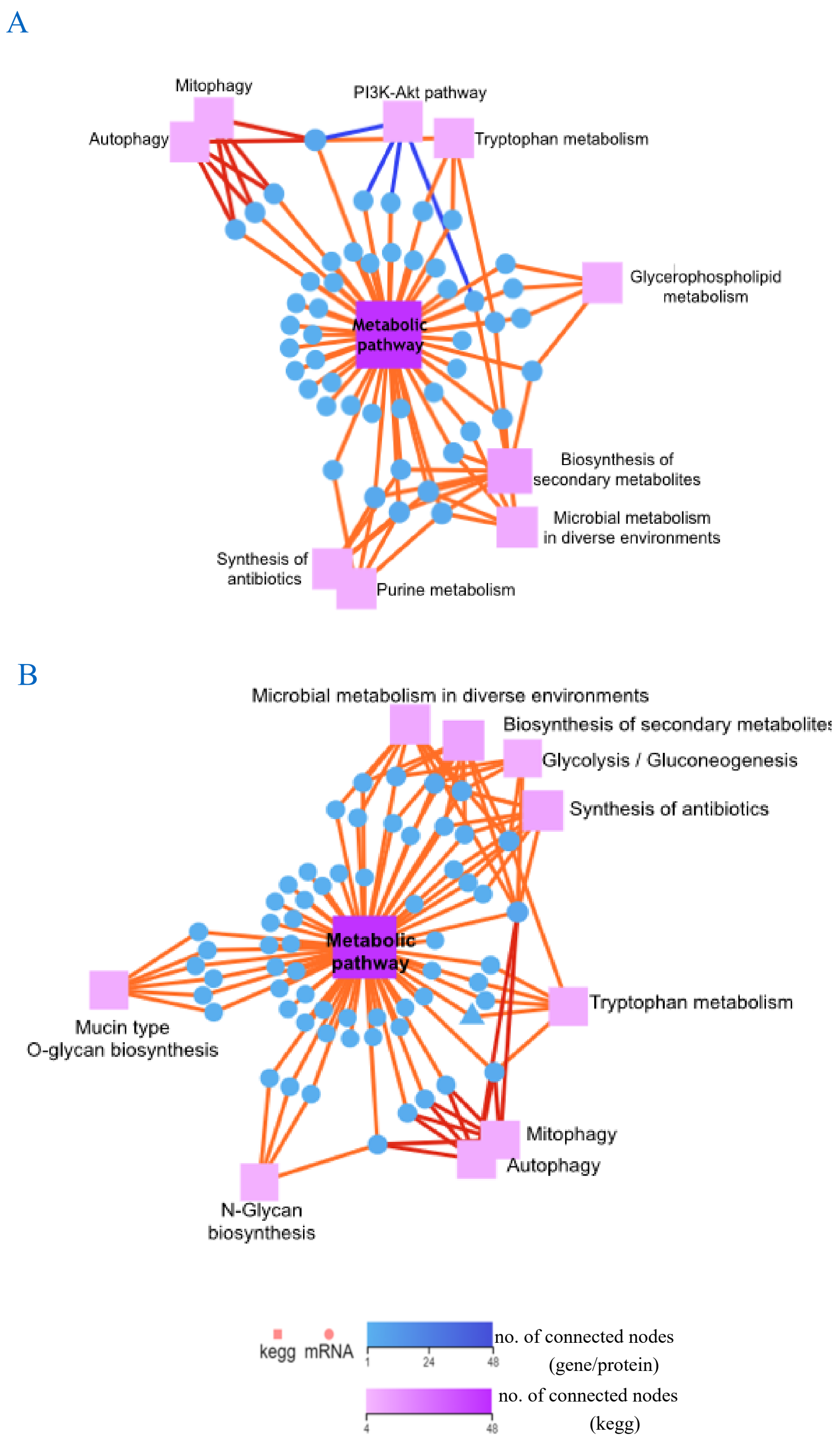

Figure S12. Correlation network of the KEGG pathways for down/regulated metabolic related pathways. Each node represents a KEGG pathway in WS5A (A) and CP2A (B) astrocytes versus control astrocytes. The blue node indicates the number of genes per protein and the pink node indicates the number of KEGG pathways.

A

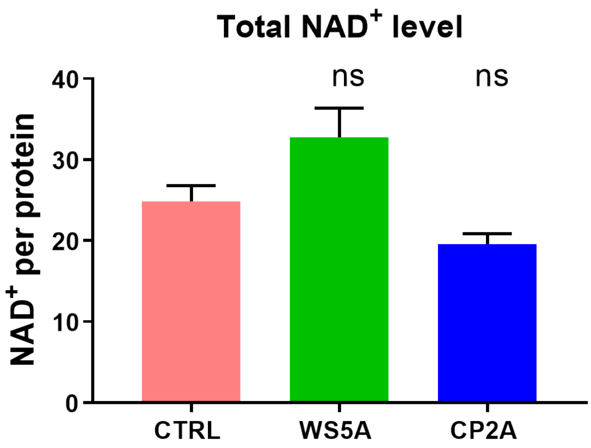

B

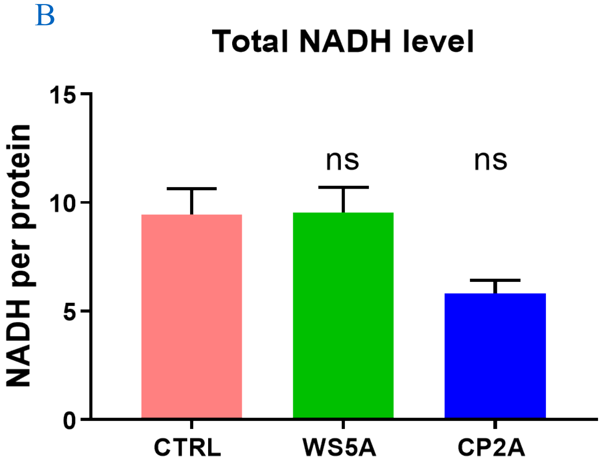

Figure S13. LC-MS-based metabolomics for quantitative measurement of total NAD^+ (A) and NADH (B).

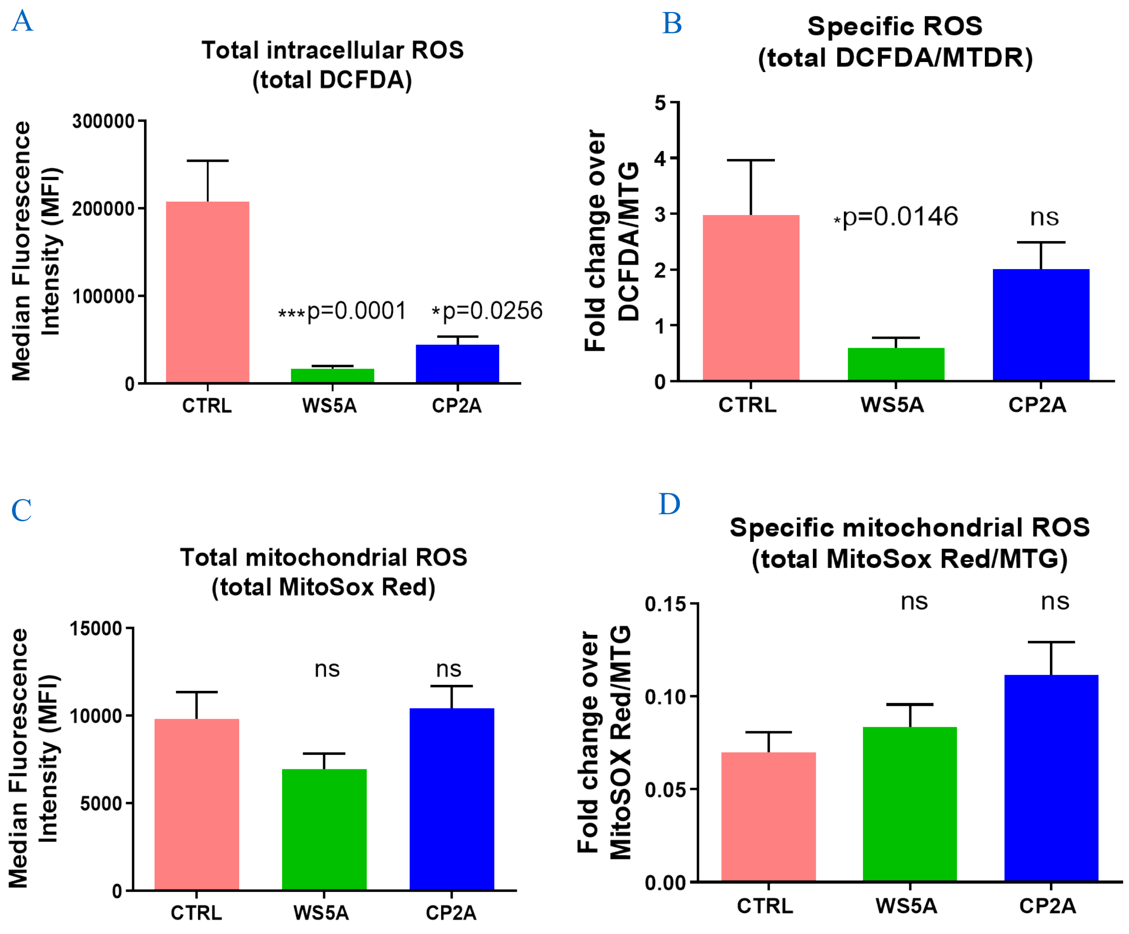

Figure S14. Flow cytometric analysis of intracellular ROS at total level (A) and specific level (B) calculated by total ROS/MTDR using double staining of DCFDA and MTDR, and mitochondrial ROS at total production (C) and specific level (D) in CTRL, WS5A and CP2A astrocytes.

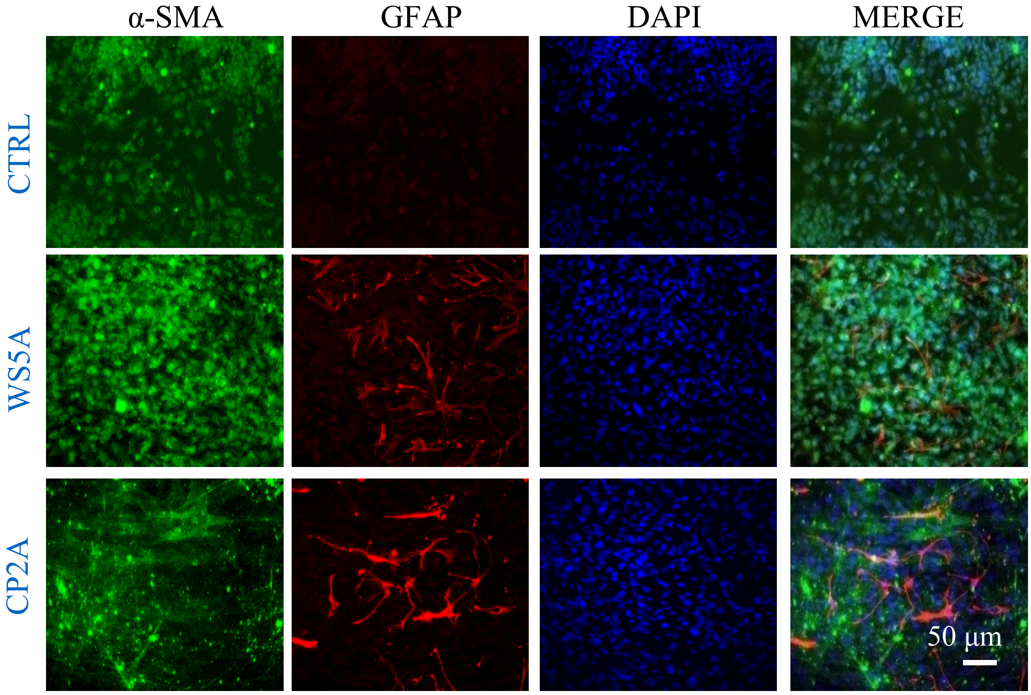

Figure S15. Representative confocal images and quantification of immunostaining of control and patient astrocytes for α-SMA (green) and GFAP (red). Nuclei are stained with DAPI (blue). Scale bar is 50 μm.

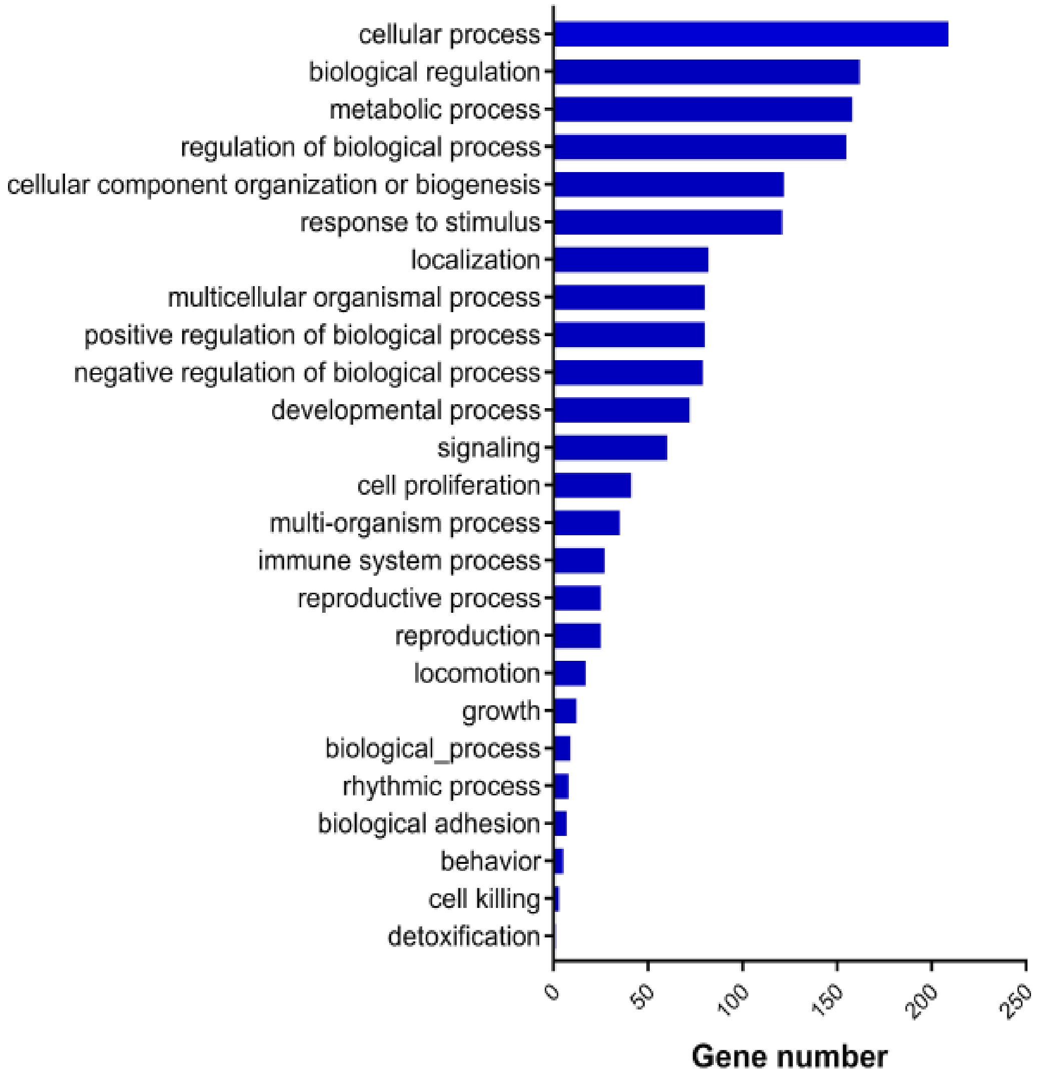

Figure S16. GO-CFP analysis of up-regulated DEGs in WS5A and CP2A astrocytes compared to CTRL astrocytes.

A

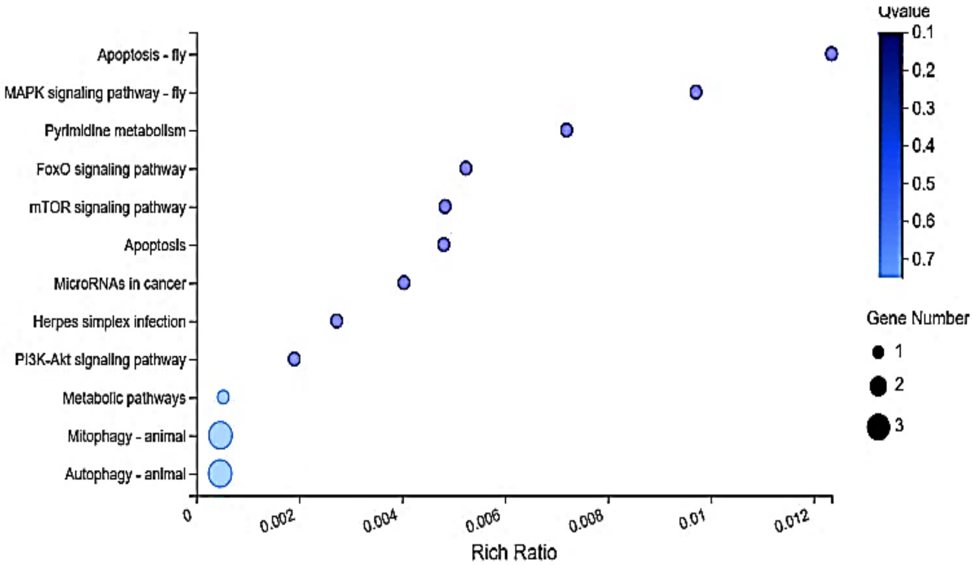

B

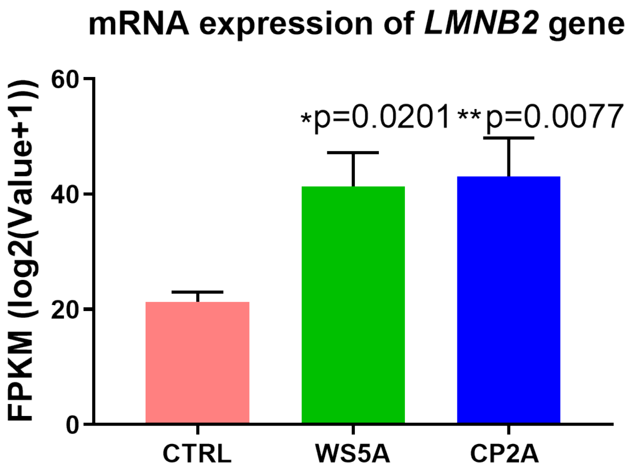

Figure S17. RNA sequencing analysis of KEGG pathway analysis (A) for the DEGs enriched in cell killing process in WS5A and CP2A astrocytes versus controls and mRNA expression for *LMNB2* gene (B).

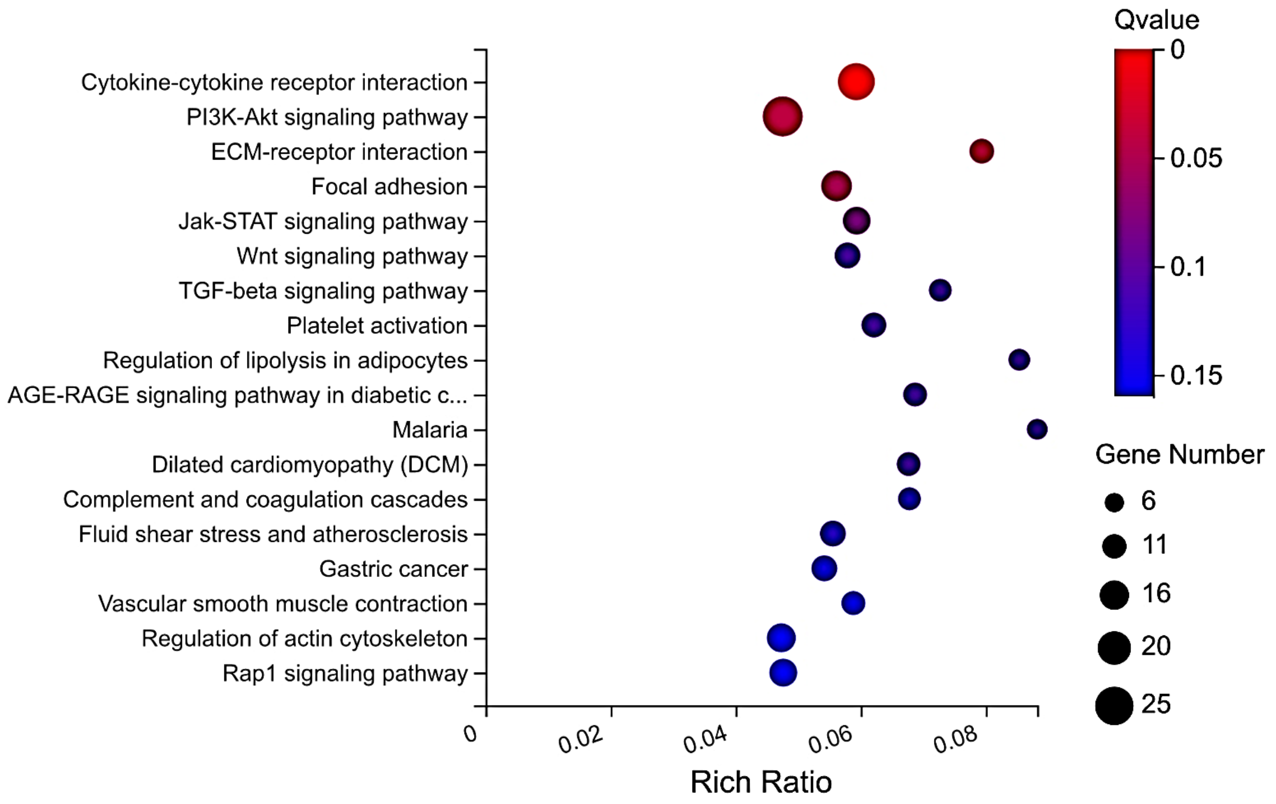

Figure S18. KEGG pathway enrichment analysis for the upregulated DEGs in patient astrocytes versus control.

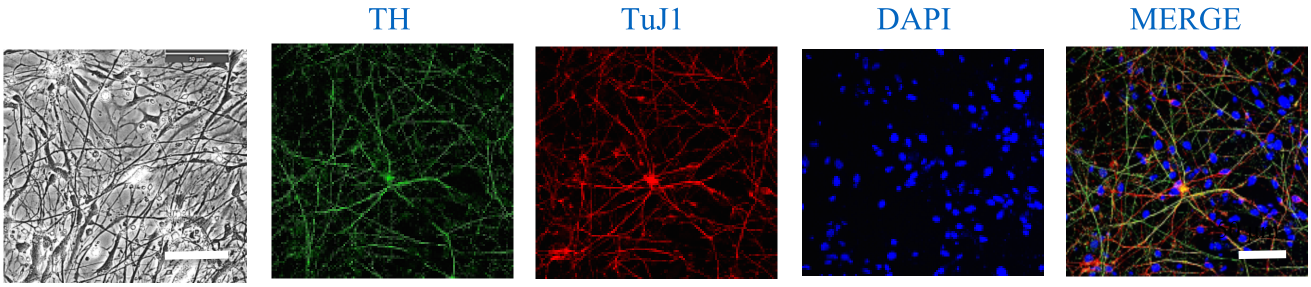

Figure S19. Representative phase-contrast and confocal images of the iPSC-derived DA neurons for immunostaining of TH (green) and TuJ1 (red). Nuclei are stained with DAPI (blue). Scale bar is 50 μm.

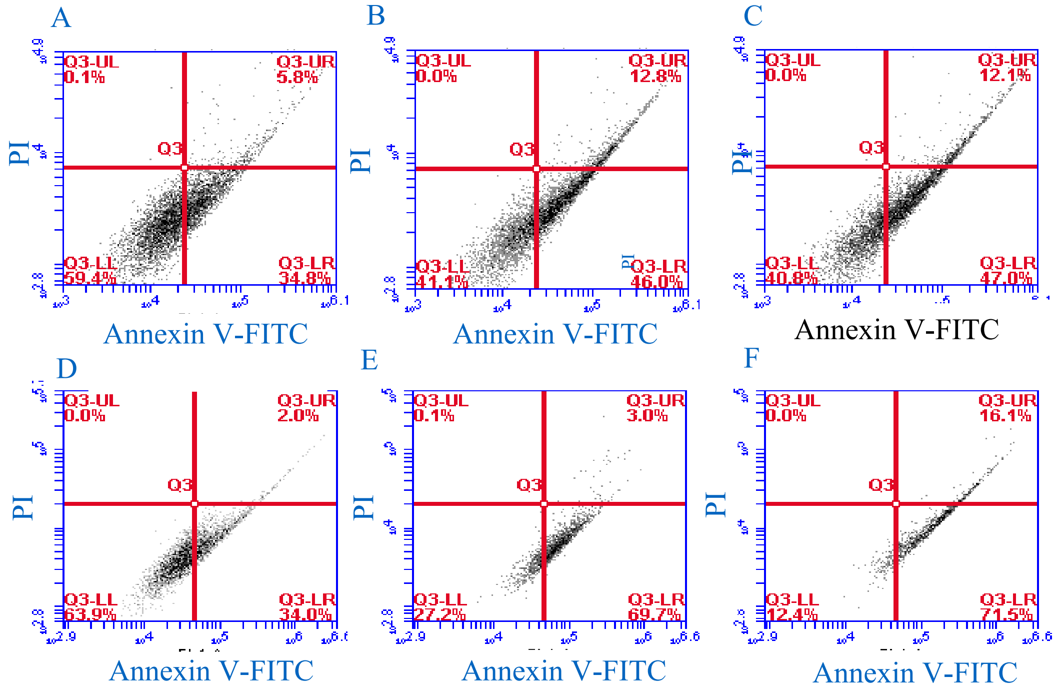

Figure S20. Representative flow cytometry plots for the percentage of pro-/early and post-/late apoptotic cells cytometry using the Annexin V-FITC/PI double staining for control (A), WS5A (B) and CP2A (C) astrocytes co-cultured with DA neurons, as well as DA neurons co-cultured with control (D), WS5A (E), and CP2A (F) astrocytes in in-direct co-culture system.
