# Supplementary material for: Activation of Neurotoxic Astrocytes Due to Mitochondrial Dysfunction Triggered by POLG Mutation
Source: Int J Biol Sci. 2024 May 11;20(8):2860–80. doi: 10.7150/ijbs.93445 (PMC11186360; doi:10.7150/ijbs.93445)
Supplement: Supplementary file 2 — Supplementary Key Resources Table. [file ijbsv20p2860s2.pdf]

Key Resources Table

REAGENT or RESOURCE	SOURCE	IDENTIFIER
Antibodies		
anti-SOX2	Abcam	Cat# ab97959, RRID:AB_2341193
anti-POU5F1	Abcam	Cat# ab19857, RRID:AB_445175
anti-PAX6	Abcam	Cat# ab5790, RRID:AB_305110
anti-NESTIN	Santa Cruz Biotechnology	Cat# sc-23927, RRID:AB_627994
anti-NESTIN-PE	R&D Systems	Cat# IC1259P, RRID:AB_2151147
anti-GFAP	Abcam	Cat# ab4674, RRID:AB_304558
anti-S100β	Abcam	Cat# ab196442, RRID:AB_2722596
anti-CD44	BD Biosciences	Cat# 555476, RRID:AB_395868
anti- α-SMA	Abcam	Cat# ab7817, RRID:AB_262054
anti-EAAT-1	Abcam	Cat# ab416, RRID:AB_304334
anti-DCX	Thermo Fisher Scientific	Cat# PA5-17428, RRID:AB_10977233
anti-GS	Abcam	Cat# ab64613, RRID:AB_1140869
anti-NDUFB10	Abcam	Cat# ab196019
anti-COX IV	Abcam	Cat# ab14744, RRID:AB_301443
anti-SDHA	Abcam	Cat# ab14715, RRID:AB_301433
anti-VDAC	Abcam	Cat# ab14734, RRID:AB_443084
anti-TOMM20	Abcam	Cat# ab56783, RRID:AB_945896
anti-GALC	Abcam	Cat# ab2894, RRID:AB_449091
anti-TH	Abcam	Cat# ab75875, RRID:AB_1310786
anti-Synaptophysin	Abcam	Cat# ab32127, RRID:AB_2286949
anti-TUJ 1	Abcam	Cat# ab78078, RRID:AB_2256751
anti-MAP2	Abcam	Cat# ab5392, RRID:AB_2138153
anti-SDHA	Abcam	Cat# ab168536, RRID:AB_2857979
anti-SDHA	Abcam	Cat# ab14715, RRID:AB_301433
anti-UCP2	Cell Signaling Technology	Cat# 89326, RRID:AB_2721818
anti-UCP2	Proteintech	Cat# 11081-1-AP, RRID:AB_2213793
anti-Phospho-SIRT1 (Ser47)	Cell Signaling Technology	Cat# 2314, RRID:AB_561516
anti-SIRT3	Cell Signaling Technology	Cat# 5490, RRID:AB_10828246
anti-beta Catenin	Abcam	Cat# ab32572, RRID:AB_725966
anti-N Cadherin	Abcam	Cat# ab76011, RRID: A_1310479
anti-GAPDH	Abcam	Cat# ab8245, RRID:AB_2107448
anti-C3	Abcam	Cat# ab97462, RRID:AB_10679468
anti-Alexa Flour® 488	Thermo Fisher	Cat# A-11008, RRID:AB_143165

	Scientific	
anti-Alexa Flour®594	Thermo Fisher Scientific	Cat# A-11005, RRID:AB_141372
anti-Alexa Flour®594	Thermo Fisher Scientific	Cat# A-11042, RRID:AB_2534099
Chemicals, Peptides, and Recombinant Proteins		
DAPI	Thermo Fisher Scientific	P36962
MTG	Invitrogen	M7514
TMRE	Abcam	ab113852
FCCP	Abcam	ab120081
DCFDA	Abcam	b11385
MTDR	Invitrogen	M22426
Mito-SOX™ Red	Invitrogen	M36008
Critical Commercial Assays		
Lactate Colorimetric/Fluorometric Assay Kit	Abcam	Cat# ab65331
Software and Algorithms		
SPSS Statistics 25	IBM	https://www.ibm.com/
GraphPad Prism version 8 for Windows	GraphPad Software, Inc	https://www.graphpad.com/
C6 Plus Workstation Computer and Software	BD Biosciences	https://www.bdbiosciences.com/us/instruments/research/cell-analyzers/bdaccuri/bd-accuri-c6-plus-options/c6-plusworkstation-computer-andsoftware/p/661391
Image J software	NIH	https://imagej.nih.gov/ij/index.html
ChromasPro DNA sequence software	Technelysium Pty Ltd	http://technelysium.com.au/wp/chromaspro/
Image Lab Software	Bio-Rad	https://www.bio-rad.com/en-us/product/image-labsoftware?ID=KRE6P5E8Z
